# Stage-dependent piRNAs in chicken implicated roles in modulating male germ cell development

**DOI:** 10.1186/s12864-018-4820-9

**Published:** 2018-06-01

**Authors:** Kai-Wei Chang, Yen-Tzu Tseng, Yi-Chen Chen, Chih-Yun Yu, Hung-Fu Liao, Yi-Chun Chen, Yu-Fan Evan Tu, Shinn-Chih Wu, I-Hsuan Liu, Marina Pinskaya, Antonin Morillion, Bertrand Pain, Shau-Ping Lin

**Affiliations:** 1 Genome and Systems Biology Degree Program, National Taiwan University, Taipei, 106 Taiwan; 2 Institute of Biotechnology, National Taiwan University, Taipei, 106 Taiwan; 3 Department of Animal Science and Technology, National Taiwan University, Taipei, 106 Taiwan; 4 ncRNA, epigenetic and genome fluidity, Institut Curie, Centre de Recherche, CNRS UMR 3244, PSL Research University, Université Pierre et Marie Curie, F-75005 Paris, France; 5 Univ Lyon, Université Lyon 1, INSERM, INRA, Stem Cell and Brain Research Institute, U1208, USC1361, F-69500 Bron, France; 6 Research Center for Developmental Biology and Regenerative Medicine, National Taiwan University, Taipei, 106 Taiwan; 7 Agricultural Biotechnology Research Centre, Academia Sinica, Taipei, 106 Taiwan; 8 Center for Systems Biology, National Taiwan University, Taipei, 106 Taiwan; 9 Present Address: Graduate Institute of Brain and Mind Sciences, College of Medicine, National Taiwan University, Taipei, 10051 Taiwan

**Keywords:** Germ cell, Chicken, piRNA cluster, Development, Transposable elements

## Abstract

**Background:**

The PIWI/piRNA pathway is a conserved machinery important for germ cell development and fertility. This piRNA-guided molecular machinery is best known for repressing derepressed transposable elements (TE) during epigenomic reprogramming. The extent to which piRNAs are involved in modulating transcripts beyond TEs still need to be clarified, and it may be a stage-dependent event. We chose chicken germline as a study model because of the significantly lower TE complexity in the chicken genome compared to mammalian species.

**Results:**

We generated high-confidence piRNA candidates in various stages across chicken germline development by 3′-end-methylation-enriched small RNA sequencing and in-house bioinformatics analysis. We observed a significant developmental stage-dependent loss of TE association and a shifting of the ping-pong cycle signatures. Moreover, the stage-dependent reciprocal abundance of LINE retrotransposons, CR1-C, and its associated piRNAs implicated the developmental stage-dependent role of piRNA machinery. The stage dependency of piRNA expression and its potential functions can be better addressed by analyzing the piRNA precursors/clusters. Interestingly, the new piRNA clusters identified from embryonic chicken testes revealed evolutionary conservation between chickens and mammals, which was previously thought to not exist.

**Conclusions:**

In this report, we provided an original chicken RNA resource and proposed an analytical methodology that can be used to investigate stage-dependent changes in piRNA compositions and their potential roles in TE regulation and beyond, and also revealed possible conserved functions of piRNAs in developing germ cells.

**Electronic supplementary material:**

The online version of this article (10.1186/s12864-018-4820-9) contains supplementary material, which is available to authorized users.

## Background

Primordial germ cells (PGCs) experience genome-wide epigenetic reprogramming for acquiring germ cell-specific features, such as meiosis, spermatogenesis and oogenesis, and regaining zygotic totipotency upon fertilization [1, 2]. This process is accompanied by burst expression of transposable elements (TEs), primarily autonomous retrotransposons such as long interspersed nuclear elements (LINEs) and long terminal repeats (LTRs) [3, 4]. The activation of transposable elements and their capability of insertion into the host genome through random transposition can lead to epigenomic and genomic instability [5].

The PIWI/piRNA pathway is evolutionarily adapted for effective mitigation of burst TE transcripts from reprogramming and is essential for proper germ cell development and fertility [6–8]. PiRNAs, namely, PIWI-interacting RNAs, are germ cell-enriched small RNAs that bind to PIWI protein and form piRNA-induced silencing complexes (piRISCs). Studies in *Drosophila* showed that the PIWI/piRNA pathway is critical for regulating TE activities in developing germ cells [6, 9]. In mice, defects in the PIWI/piRNA pathway result in aberrant expression of TEs that leads to germ cell depletion and subsequently small testes and infertility [10–13]. Knockdown of the chicken PIWI proteins, CIWI and CILI, also leads to an upregulation of chicken LINEs – chicken repeat 1 (CR1) elements, and hence supports the conservation of the PIWI/piRNA pathway in TE repression [14, 15].

The molecular mechanisms by which piRNAs modulate TEs are partly implicated through their biogenesis pathway. The primary piRNA precursor transcripts from piRNA clusters are transported to the perinuclear electron-dense region, the so-called nuage structure, for the maturation process [16]. The 5′ end of a piRNA is generated through MITOPLD (in mice)/Zuc (in *Drosophila*) cleavage and loaded onto the PIWI nucleotide binding pocket in 5′ uracil (1 U) preference fashion [16, 17]. The *Drosophila* Nibbler, or PARN-family exonucleases in other species, are reported to be involved in trimming the 3′ ends to form 24–32 nt small RNA fragments [18–20], which then have their 3′-end modified by 2’-O-methylation via HEN1 and form primary piRNAs [21–23]. Mature piRISCs identify transcripts antisense to their piRNA sequences and slice the targeted transcripts by the endonuclease function of PIWI protein at the position corresponding to the 10th nucleotide of piRNA [24, 25]. The cleaved transcript fragments are bounded by other PIWI proteins, such as MIWI, MILI, and MIWI2 in mice, and are then processed into antisense piRNAs, which also form piRISCs capable of slicing other transcripts [26]. This ping-pong cycle machinery of looped sense-antisense targeting, which is mainly processed via MILI in mice, can rapidly amplify secondary piRNAs by consuming TE transcripts [9, 27, 28]. Due to the preferred U for the first nucleotide of primary piRNAs and reverse complementary targeting over the ping-pong cycle, these secondary piRNAs feature the enrichment of adenine at the 10th nucleotide (10A) [27, 29]. Comparatively, *Drosophila* AGO3 and AUB participate in a ping-pong cycle in which they, respectively, bind with sense and antisense TEs [9, 30, 31]. PiRISCs composed of certain PIWI family members, such as MIWI2 in mice or PIWI in *Drosophila*, can also transport cytoplasmic piRNAs into the nucleus and mediate epigenetic gene silencing through H3K9 di- or tri-methylation and euchromatic de novo DNA methylation [32–37]. Hence, piRNAs may operate via post-transcriptional gene silencing (PTGS) and transcriptional gene silencing (TGS) to modulate TE expression and possibly beyond.

The emerging evidence implies diverse roles of the PIWI/piRNA pathway along germ cell development in a stage-dependent fashion. For instance, changes in piRISC composition were reported along the different mouse germ cell developmental stages [38]. The conditional inactivation of the *Miwi2* gene revealed that MIWI2 is essential for prospermatogonia development in mice [39]. In contrast, MIWI is expressed and involved in ping-pong cycle-independent TE silencing after birth [10, 29]. PiRNA cluster analysis of MILI-interacting piRNAs showed distinct genomic associations, from the pre-pachytene TE-rich piRNA population and pachytene intergenic piRNA population [26]. Moreover, a recent study demonstrated the switching of dominant TE silencing machinery from the PIWI/piRNA pathway in spermatogonia to DNA methylation in meiotic spermatocytes [40], which is a further indication of stage-dependent regulation in the PIWI/piRNA pathway. Here, we extend our investigation to the roles of piRNAs along different germ cell developmental stages.

Since the development of the chicken embryo can be synchronously controlled and the chicken embryo developmental stages are well documented [41–43], chicken is a suitable model organism for studying stage-dependent effects of conserved machinery, such as stage-associated piRNA regulation. In addition, TEs constitute less than 10% of the chicken genome, which is significantly lower than TE occupancies in other tetrapod vertebrates, such as 48% in the human genome (hg38) and 41% in the mouse genome (mm10) [44–47]. Chicken TEs are also less complex than their mammalian counterparts. Moreover, chicken serves as an important evolutionary model for the conservativity of the PIWI/piRNA pathway. Together, these factors make chicken a plausible model for analyzing the roles of piRNAs in and potentially beyond TE modulation throughout different developmental stages. The expression of PIWIL1 in chicken PGCs has been reported [48]. In addition, the presence of piRNAs has been reported in chicken PGCs and adult testes [15, 49, 50]. Nevertheless, the stage-dependent expression and genomic association of chicken piRNAs has yet to be systematically analyzed.

In this study, we performed an in-depth analysis of piRNA clusters based on 3′-end-2’-O methylation-enriched small RNA sequencing on germ cells taken at different developmental stages. Here, we show the stage-dependent transition in piRNA compositions and their roles in TE regulation. Our in-depth investigation of piRNAs before and after spermatogonia formation reveals that piRNA-associated TE regulation may also contribute to gene regulations. In extension, our results display the stage-dependent expression of some genomic loci embedding putative piRNA clusters. We further investigated a functional implication of these stage-dependent clusterable piRNAs and propose a possible role of PIWI/piRNA pathways in germ cell fate decision.

## Results

### Implementation of a computational workflow for high-confidence piRNA discovery

To identify piRNA candidates among gonadal small RNA pools, we applied sodium periodate oxidation combined with small-RNA high-throughput sequencing (small RNA-seq) and in-house developed computational workflow dedicated to the identification of piRNA candidates (Additional file 1: Figure S1). In vertebrates, only piRNAs but not miRNAs contain a 3′-end 2’-O-methyl modification, which resists oxidation treatment and remains ligatable at the 3′-end for sequencing library construction [51]. This 3′-end 2’-O-methylation signature has been observed in chicken piRNAs [49], thus supporting the applicability of this method in our study. Reads from the oxidized small RNA libraries were then processed through our bioinformatic pipeline. Our implementation of the bioinformatics pipeline aimed to identify potential piRNA reads by eliminating other forms of small RNAs instead of considering only TE-derived reads [52]. Briefly, after genomic mapping, reads that annotated to rRNA, tRNA, and miRNA were removed. To ensure the elimination of false positives, we also eliminated reads that may have originated from predicted microRNA precursors.

Given the expression of chicken PIWI/piRNA pathway-associated genes found in blastoderm [48, 52], cultured PGC [15, 48], and adult testes [49], we validated that these genes are also expressed in enriched embryonic germ cells around spermatogonia formation stages (Additional file 1: Figure S2, S3). Our in-house bioinformatic pipeline was then applied over 3′-end-2’O methylation-enriched small RNA-seq datasets from blastodermal cells (BC), cultured PGCs from E7 gonads (E7PGC), and E11 and E14 embryonic gonads (E11G and E14G, respectively) generated in our lab. PiRNA datasets of adult testes (AT) published by Li et al. [49] were included to evaluate the accuracy of our piRNA candidate analysis pipeline and provide a piRNA dataset of germ cells undergoing meiosis. The small RNA categories and read densities through each step of filtering and final piRNA candidacy are summarized in Additional file 1: Table S1. The high proportion of mapped reads was categorized as piRNA candidates based on our analysis pipeline predictions (Additional file 1: Figure S1C). The reduction of reads mapping to miRNAs in the oxidation-treated libraries compared to the untreated counterparts demonstrated the success of enriching 3’end modified small RNAs.

**Fig. 1:**
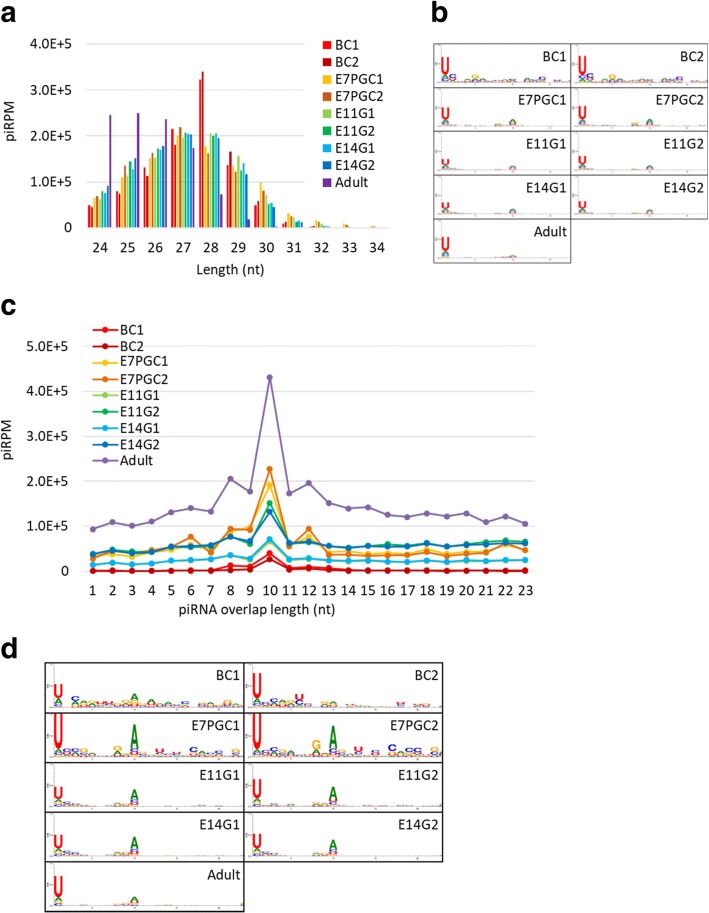
PiRNA candidates show distinct piRNA features across different developmental stages. **a** Length distribution of piRNAs. PiRNA reads per million (piRPM) is calculated for each read length; **b** Nucleotide enrichment analysis on piRNA candidates in each sequencing sample. **c** Relative enrichment of pairable piRNAs by the overlapping length. PiRPM is calculated for the number of pairable piRNAs with each overlapping length. **d** Nucleotide enrichment analysis of piRNA candidates of 10 bp antisense overlapping at the 5′ end in each sequencing sample

The characterization analysis results showed that the adult testicular piRNA candidates are shorter in length, mostly ranging from 24 to 27 nt, relative to piRNA candidates from other samples, which range from 26 to 29 nt (Fig. 1a). We further validated the piRNA candidates in each sample with known piRNA characteristics. The resultant piRNA candidates showed strong 5′-end uracil (1 U) enrichment (Fig. 1b), which supported the 1 U-bias of piRNAs due to structural preferences of the nucleotide binding pocket of the PIWI MID domain [53]. In addition, we investigated the ping-pong cycle activities, which are characterized by a frequent mutual overlap of 10 nt at the 5′-end and 1U10A enrichment. We observed frequent 5′-10 nt overlapping (Fig. 1c; Additional file 1: Figure S4) relative to other 5′ overlapping lengths. However, the low sequence overlap rate among embryonic samples implied limited ping-pong cycle activities. The nucleotide enrichment analysis of 5′-10-nt overlapping piRNAs showed a 10A bias in samples other than BC. Interestingly, we observed a gradual loss of 10A feature along germ cell development (Fig. 1d). This observation supported stage-dependent variations in piRNA composition.

**Fig. 2:**
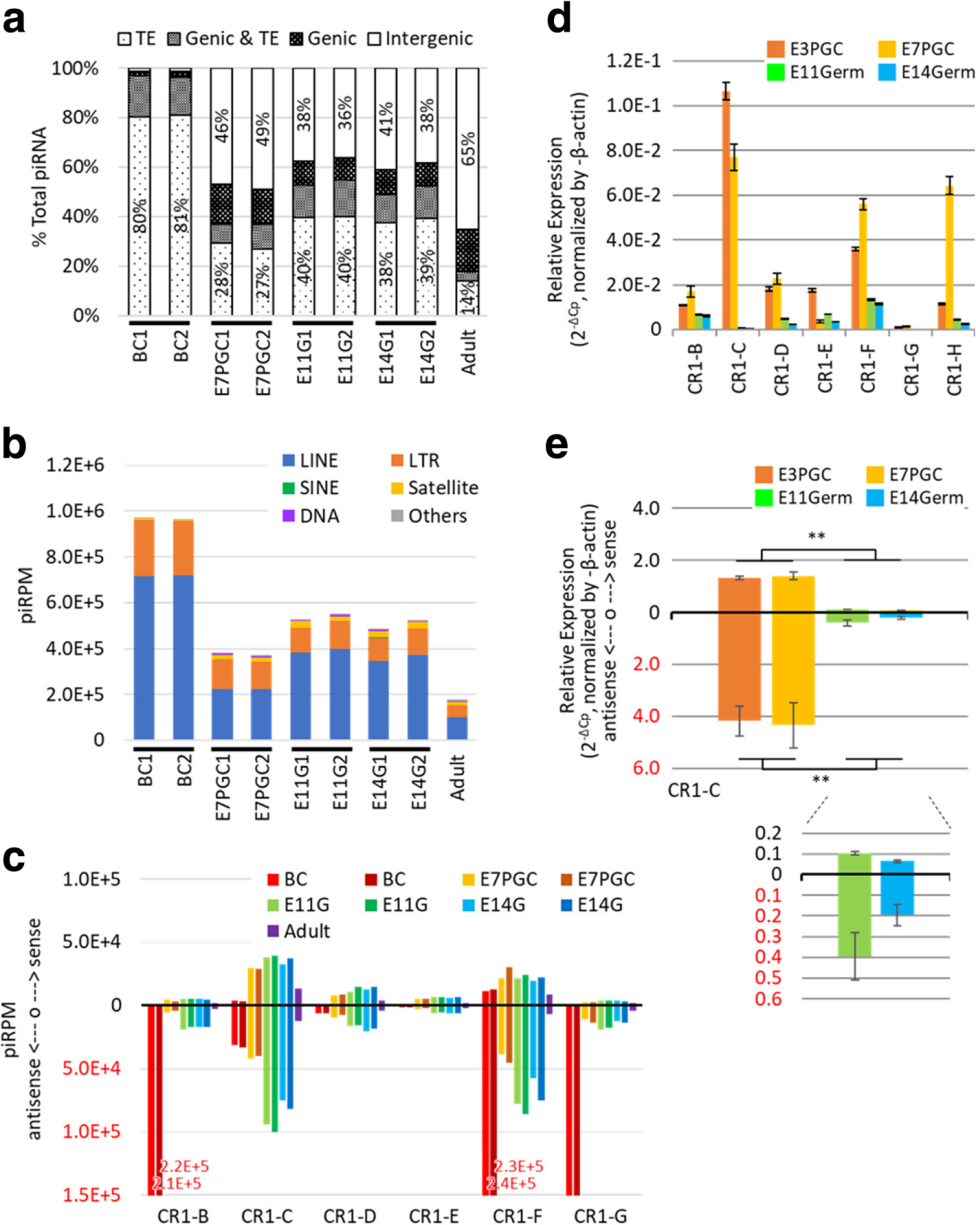
Developmental stage-dependent genomic associations and TE expression modulation of piRNA candidates. **a** Genomic association varied in stage-dependent fashion. Each feature was calculated in proportion to the respective total piRNA candidates. **b** PiRNA candidates mapped to TEs. piRPM is calculated for each enlisted category. **c** The stage-dependent association of piRNAs to subfamilies of LINEs. PiRNA sequences are preferentially mapped antisense to TEs. **d** Expression of CR1 subfamily members from enriched embryonic germ cells. **e** Stranded RT-qPCR analysis (*N* = 3) over CR1-C transcription among cultured E3 circulating PGC (E3PGC), cultured E7 gonadal PGC (E7PGC), and germ cell enriched population, E11Germ and E14Germ, from E11 and E14 Gonads, respectively. ** represents *p*-value < 0.01

Remarkably, over 90% of the piRNA candidates from BC mapped to TEs, and less than 5% mapped to intergenic regions. In contrast, adult testes showed significantly higher intergenic association over 65% (Fig. 2a). A reduced TE association of piRNA candidates was also observed in cultured E7PGC, E11G and E14G, compared to that from BC. PiRNA candidates from cultured E7PGC had almost 50% of their reads mapped to intergenic regions, but only approximately 35% of the piRNA mapped to TEs. These observations suggested dynamic regulation of genomic associations of piRNAs toward the prevalence of intergenic loci along germ cell development. While the chicken PIWI/piRNA pathway may encompass stage-dependent biosynthesis machineries favoring production of piRNAs in a certain length range [54], we observed no significant correlation between piRNA length distribution and genomic association in a stage-dependent manner (Additional file 1: Figure S5).

### PiRNA candidates demonstrated stage-dependent association to TE subfamilies

Given the involvement of piRNAs in TE regulation [55], their reduced TE association in a stage-dependent manner may be due to TE repression (Fig. 2a, b). This effect may be more prominent for piRNAs assigned to LINEs (Fig. 2c). Expression analysis by RT-qPCR of a purified embryonic chicken germ cell population generally showed a downregulation of LINE components between PGCs and enriched germinal cells at late embryonic stages (Fig. 2d, e). Notably, we observed the dynamic expression of LINEs from the enriched germ cell populations among cultured PGCs from E3 and E7 and freshly isolated germ cells from E11G and E14G. This implies that TE expression may also be regulated in a stage-dependent manner. Indeed, we further observed stage-dependent reciprocal correlations between the expression of LINE members and the abundance of their corresponding piRNAs (Fig. 2c-e). Among the transcriptionally repressed LINE members CR1-C, CR1-F, and CR1-H, there were more LINE associated piRNAs mapped antisense to LINE, than the piRNAs mapped to the sense direction, by 2 to 5 fold. Intriguingly, we observed a high abundance of antisense piRNAs associated with CR1-B, CR1-F, and CR1-G, in accordance with the high repressive strength at BC. In contrast, we observed that the expansion of piRNAs associated with CR1-C occurred later, at E11 (Fig. 2c). Strand-specific RT-qPCR on CR1-C showed a downregulation of both sense and antisense transcripts at E11G and E14G. In addition, both PGCs and gonadal germ cells showed higher levels of antisense transcripts, and this finding supported the positive correlation with the associated antisense piRNAs (Fig. 2d, e). These results indicate that the PIWI/piRNA pathway may have a role in the stage-dependent transient expression of TEs.

### Investigating the possibility of piRNA-mediated modulation beyond TEs in chicken embryonic gonads

To evaluate the possible contribution of the developmentally regulated piRNAs in modulating transcriptome beyond TEs, we first tested the hypothesis that differentially expressed piRNAs between E11G and E14G gonads may be involved in modulating spermatogonia formation, which was estimated to occur around E13 [43]. Approximately 20% of piRNA candidates from E11G and E14G were mapped to either the sense or antisense strand of genes/transcripts, in which half of these piRNA-associated transcripts were not TE-associated (Fig. 2a). This finding suggested that piRNAs may have roles beyond but not mutually exclusive with TE regulation.

**Fig. 3:**
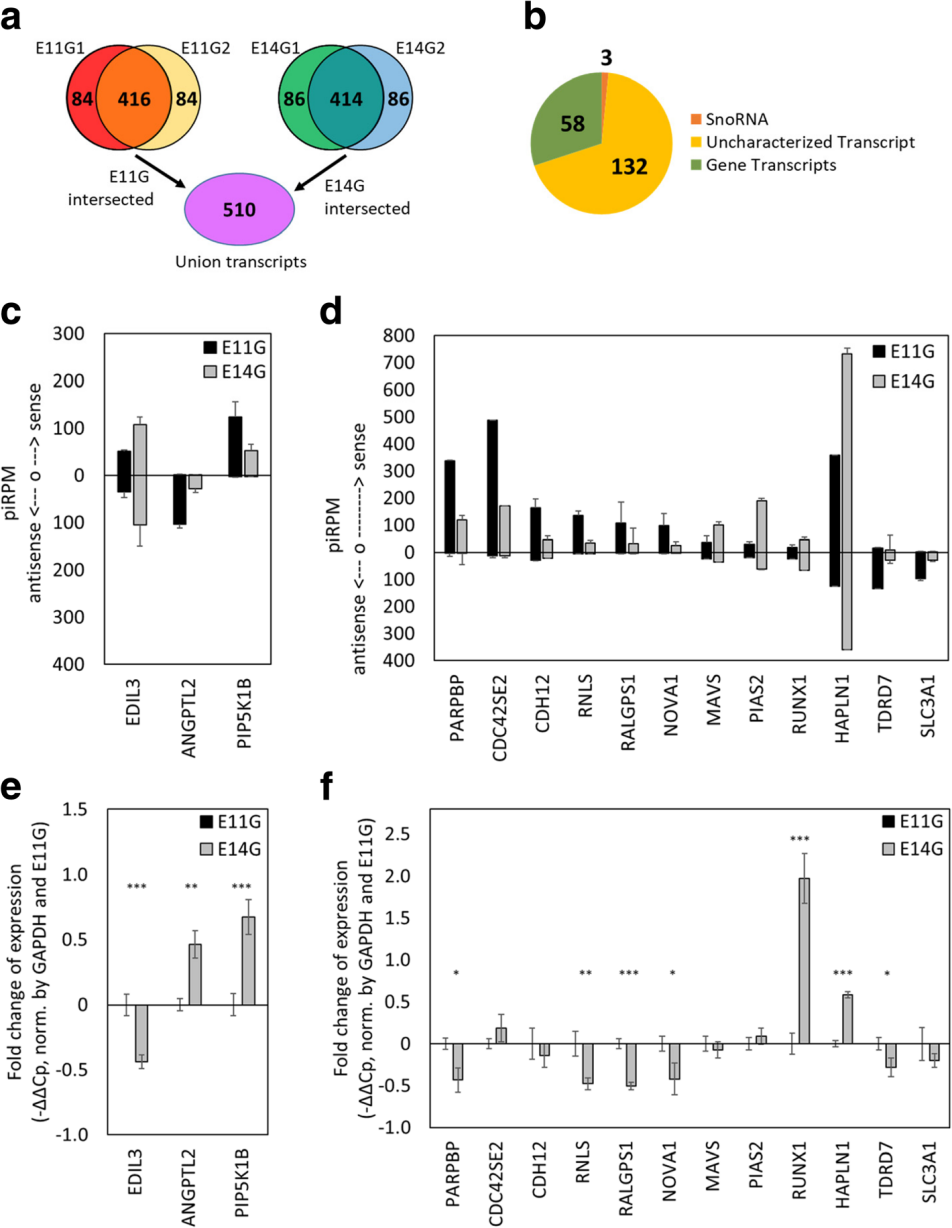
Identification of potential piRNA associated genes before (E11G) and after (E14G) spermatogonia formation. **a** Venn diagram of piRNA-associated target transcripts before and after spermatogonia formation. The top 500 target transcripts were selected from each sample, and the union of reproducible piRNA associated transcripts from E11 and E14 gonads revealed 510 potential piRNA modulated transcripts. **b** Transcripts with 2-fold difference in normalized piRNA counts are identified, of which 58 are annotated genes. **c**-**d** PiRNAs mapped to the sense or antisense of their associated genes. **e**-**f** Comparison of relative expression levels of potential piRNA associated genes between enriched germ cells from E11 and E14 gonads (*N* = 3). *represents *p*-value < 0.05; **represents *p*-value < 0.01; ***represents *p*-value< 0.001. Five genes are reciprocally expressed compared to the amount of associated piRNAs (**e**), while 12 genes do not have reciprocal relative expression levels to the numbers of piRNAs mapped to them (**f**)

Among the top 500 piRNA-associated transcripts heavily mapped by piRNAs (with at least 70 piRPM) for E11G and E14G of both Leghorn layer and Cobb500 broiler breeds, we selected the 416 and 414 overlapped genes from the two chicken strains of E11 and E14 testes, respectively. We found 510 transcripts that were potential piRNA associated transcripts of E11G and E14G in union (Fig. 3a), among which 193 transcripts were differentially expressed (fold change > 2) before and after spermatogenesis (E11G vs E14G). Among these candidate transcripts, we identified 58 protein-coding genes, 132 uncharacterized genes from Ensembl, and 3 snoRNAs (Fig. 3b). However, none of the Gene Ontology (GO) terms was significantly enriched with these genes. The differential expressions of 15 of the targets between E11 and E14 germ cells were examined by RT-qPCR (Fig. 3c-f). Three of these genes showed negative correlations between gene expression and the associated piRNAs, which were predominantly mapped on or approximate to TEs within those genes (Fig. 3c, e; Additional file 1: Figure S6). Uniquely mapped piRNAs associated with the 3 candidate genes were highly represented, which indicated that even when considering the repetitive nature of the TE sequences within genes, only a small portion of piRNA reads associated with the aforementioned genes can also be mapped to the identical sequences residing on the other part of the chicken genome. The preferential TE-associated piRNA mappings were also observed in most of the other top piRNA-associated transcripts (Additional file 2: Table S2). However, due to the lack of a strong reciprocal correlation between antisense piRNA mapped reads and the expression of targeted transcripts across different developmental stages, gene silencing based on piRNA-mediated targeting and slicing may not be a common event in embryonic chicken testes. We do not exclude the possibility that some of the piRNA-associated transcripts were regulated by piRNAs via post-transcriptional slicing or piRNA-mediated epigenomic silencing.

### piRNA cluster analysis identified stage-dependent piRNA expression patterns

**Fig. 4:**
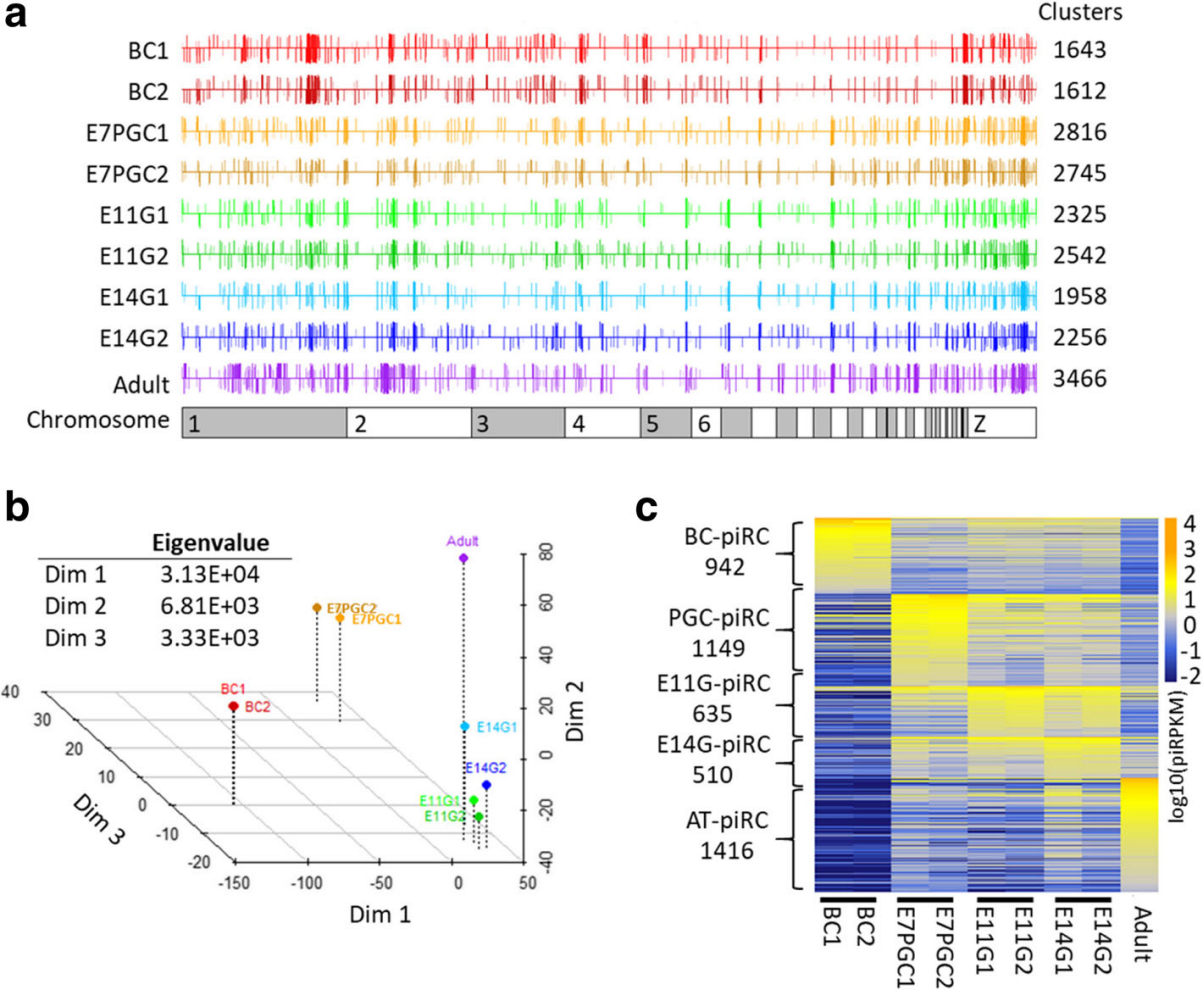
PiRNA cluster analysis reveals stage-dependent differential expression of piRNA precursors. **a** PiRNA cluster locus identified in each sample via proTRAC. Each bar represents a cluster at the plus (up) or minus (down) strand. Length and color depth denote the piRNA density in percentile. **b** Multidimensional scaling reveals the stage-associated piRNA cluster expression profile. Eigenvalues approximate the degree of variation explained by each dimension. **c** Identification of stage-enriched piRNA clusters (piRCs), each of which has piRPKM 1.5-fold higher than the second-highest stage. Stage-enriched piRNA clusters from blastodermal cells (BC), primordial germ cells (PGC), embryonic gonads from E11 and E14 (EG), and adult testes (AT) are included

To systematically illustrate developmental changes in piRNA compositions, we performed piRNA cluster analysis to identify genomic regions likely transcribing piRNA precursors (Fig. 4a). Our analysis showed that more than 70% of piRNAs can be assigned to potential piRNA clusters, with most being 3–10 kilobases in length and some reaching megabases in length (Additional file 1: Figure S7A). Cross-stage comparison of the genomic loci of piRNA clusters revealed globally similar expression patterns (Fig. 4a). However, some clusters demonstrated significant developmental stage-dependent differential piRNA production (Fig. 4a; Additional file 1: Figure S7B). Moreover, we identified a set of piRNA clusters that exhibited strict stage dependencies. The intersection of joined overlapping piRNA clusters across samples and transcriptome information allowed us to better predict the piRNA cluster boundaries (Additional file 1: Figure S8; see Material and Method). This adjustment granted a slight increase in the number of piRNA candidates that may be assigned to piRNA clusters despite being initially discarded by proTRAC, a software program for piRNA cluster detection (Additional file 1: Table S3). Multidimensional scaling analysis showed a strong association between piRNA cluster expression patterns and developmental stages (Fig. 4b). As demonstrated, the expression profiles of piRNA clusters between E11G and E14G were less distinguishable, suggesting similarities in piRNA modulations before and after spermatogonia formation. Hence, we categorized the expression profiles of piRNA clusters according to the developmental stages, as BC-enriched piRNA clusters (BC-piRC), PGC-enriched piRNA clusters (PGC-piRC), embryonic gonadal-enriched piRNA clusters (EG-piRC), and adult testes-enriched piRNA clusters (AT-piRC). We found that nearly 70% of the 7269 piRNA clusters showed stage dependency, with an expression cutoff of 0.1 piRPKM and 1.5-fold expression enrichment (Fig. 4c). Remarkably, the piRNAs that mapped to these stage-enriched piRNA clusters showed similar genomic association patterns as the mapping results from the piRNA samples: the enriched TE-associated piRNAs in BC-piRCs; the enriched intergenic-associated piRNAs in AT-piRCs; and the “in-between” piRNA pool in PGC-piRCs and EG-piRCs. The developmental stage-specifically upregulated piRNA clusters implied the existence of open genomic regions for piRNA precursor transcription that may play stage-associated regulatory roles.

### Stage-dependent chicken piRNA clusters revealed evolutionary conservation with eutherian piRNA clusters

**Fig. 5:**
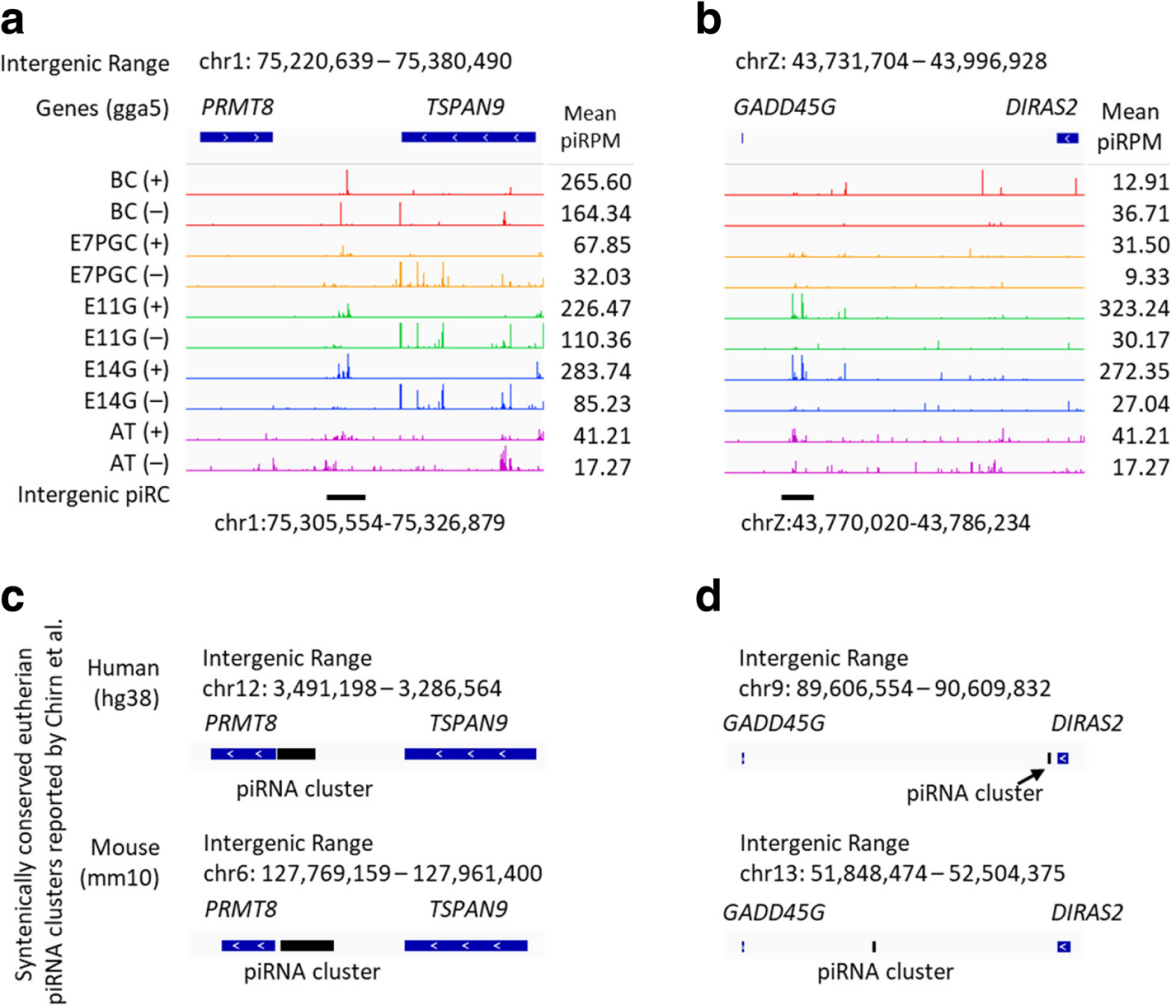
Potential stage-dependent syntenically conserved piRNA clusters shared between eutherian and chicken. The intergenic chicken piRNA cluster expressions were reported as piRPM at between (**a**) PRMT8 and TSPAN9; and (**b**) GADD45G and DIRAS2. These loci were reported syntenically conserved in eutherian by Chirn et al., and are respectively listed at (**c**) and (**d**) for comparison. Both human and mouse piRNA clusters are listed. Stage-enriched piRNA clusters from chicken blastodermal cells (BC), primordial germ cells (PGC), embryonic gonads from E11 and E14 (E11G and E14G), and adult testes (AT) are included. The novel syntenically conserved clusters between eutherian and chicken were revealed from our original piRNA datasets from embryonic gonads

The conservation of piRNA clusters between avian and eutherian mammals has not been discovered. With the newly identified chicken piRNA clusters from embryonic gonads (Additional file 1: Figure S7), we re-investigated the potential conservation of piRNA clusters with previously reported eutherian-conserved piRNA clusters [56]. We identified several syntenically conserved piRNA clusters between chickens, mice and humans, for example, the intergenic piRNA clusters residing between *PRMT8* and *TSPAN9*, and between *GADD45G* and *DIRAS2* (Fig. 5). Interestingly, the transposable elements from these two conserved clusters are very similar among the three species. Both potentially conserved chicken piRNA clusters are expressed only at E11 and E14 gonads but not in adult testes (Fig. 5). This suggests that the expression of these conserved piRNA clusters is modulated in a developmental stage-dependent manner in chickens.

### piRNAs from E11 and E14 gonad-enriched piRNA clusters preferentially targeting neural lineage-associated genes

The stage-dependent expression of piRNA precursors from each cluster likely contributes to the changes in piRNA composition during germ cell development. We found that stage-enriched piRNA cluster-derived piRNAs targeted similar TEs, which are predominantly CR1 and ERVL elements (Additional file 3: Table S4). On the other hand, these different stage-enriched piRNAs mapped antisense to a large variety of genes (Additional file 1: Figure S9). We then applied GO enrichment analysis via Enrichr [57] using the piRNA-targeted transcripts for potential functional annotations. Surprisingly, the genes targeted by EG-piRC piRNAs were predominantly expressed in the brain, based on the Mouse Gene Atlas category [57], and were primarily associated with synaptic transmission and neural development based on our gene ontology enrichment analysis (Additional file 1: Figure S10, S11; Additional file 3: Table S4). From the gene list associated with GO term: “positive regulation of nervous system development (GO:0051962)”, we randomly selected several EG-piRC piRNA-targeted genes for expression analysis via RT-qPCR on enriched germ cells of different developmental stages. We found that the expression of these genes decreased in germ cell-enriched samples at E14 gonads compared with earlier developmental stages (Additional file 1: Figure S12). Further investigation revealed that piRNAs associated with these genes were predominantly mapped to the TEs embedded within the gene bodies. Since there was a significant representation of uniquely mapped piRNAs to these TE regions, any potential biases resulting from multiple mapping due to identical sequences have been minimized. Nevertheless, whether a specific subset of TE-embedded genes can indeed be modulated by piRNAs and cause significant biological consequences relies on further functional analysis.

## Discussion

### PIWI/piRNA pathway in chicken and mouse

PIWI family members are known for having stage-specific expression in mouse germline development [10, 33]. *Miwi*, also known as *Piwil1*, is expressed from the pachytene stage of meiosis to the haploid round spermatid; *Mili*, also known as *Piwil2*, is expressed from PGCs to the round spermatid; and *Miwi2*, also known as *Piwil4*, is expressed mainly in quiescent prospermatogonia. However, only the *Piwil1* and *Piwil2* homologous genes, *CIWI* and *CILI*, and not *Piwil4* are annotated in the chicken genome [14]. In this study, the RNA transcripts of *CIWI* and *CILI* were detected in male embryonic gonads after sex differentiation (E8) to spermatogonial formation (E14). *CIWI* and *CILI* transcripts were reported to be detectable in PGCs and adult gonads [14]. These data supported the existence of PIWI/piRNA pathways along chicken germ cell development but also indicated that, unlike in mice, chicken PIWI family genes likely do not exert stage-specific functions.

However, the variations in the length distribution of piRNA candidates, and the gradual loss of the 10A feature conservation in piRNA candidates involved in the ping-pong cycle along germline development, supported the possibility that distinct PIWI/piRNA pathway machineries operate in a stage-dependent manner. Moreover, the dominant expression of *CIWI* from E8G to E14G further implied roles of CIWI in the chicken PIWI/piRNA pathway (Additional file 1: Figure S3). Whether components within a PIWI complex affect PIWI-piRNA binding and processing machineries remains elusive. Our results showed the upregulation of PIWI/piRNA-associated genes, which may be associated with the stage-dependent co-factor composition within PIWI complexes.

### Comparison of the piRNA composition along germ cell developmental timelines between chicken and mouse

Our piRNA-seq analysis revealed changes in piRNA population among BC, cultured E7 PGC, E11 and E14 gonads, and adult testes, which, respectively, correspond to PGC precursors, gonadal PGCs, prospermatogonia, spermatogonia, and mature germ cell population. Since galGal5 chicken genome assembly came from red jungle fowl (origin of domesticated chicken), our “no-mismatch” piRNA candidates analysis pipeline identified piRNA sequences of the wildtype chicken piRNAs prior domestication. We found that the piRNA composition changed from highly TE-associated BC piRNAs to predominantly intergenic region-associated adult testicular piRNAs. Comparatively, emerging studies in mouse germ cell piRNAs have implied a distinctive border for piRNA compositions, which are predominantly TE-associated pre-pachytene piRNAs and predominantly intergenic orientated pachytene piRNAs [13, 58–60]. We observed a similar reduction of TE-associated piRNAs from early to late germ cell development between chickens and mice. Since the PIWI/piRNA pathway may contribute to epigenetic silencing via H3K9me3 and DNA methylation onto the piRNA targeted regions [33, 61], piRNAs expressed in primordial germ cells may silence their host transcripts and may thus reduce the transcription activities from the same region in later germ cells. Interestingly, however, piRNAs from cultured E7PGC and gonads from E11 and E14 showed “in-between” genomic associations with similar representation of TEs and non-TE loci. Since chicken male germ cells enter meiosis after sex maturation approximately 10 weeks after hatching [43], the transition of chicken piRNA compositions from TE-targeting piRNAs to intergenic-associated piRNAs in male germ cells might be a progressive event. Moreover, our analysis showed a significant number of intergenic gonadal piRNAs before entering meiosis, suggesting differences in piRNA-associated TGS machineries between chickens and mice.

It is curious that our piRNA candidate analysis pipeline identified the presence of rRNA and tRNA derived small RNAs at early embryonic stage from the 3′-end modified population after periodate oxidation treatment (Additional file 1, Figure S1). The low number of microRNAs in our 3′-end modification enriched population confirmed the success of oxidation treatment (Additional file 1: Figure S1C, Non-enriched vs. Oxidation Enriched). Our data suggests the presence of rRNAs and tRNAs editing, including methylation. We haven’t been able to obtain direct evidence for 3′-end modification in rRNAs and tRNAs in this study, but 2’-O methylation modifications on rRNA and tRNA have been reported in eukaryotes, including yeast and human. Additionally, other methylation editing may also block the oxidation reaction. The RNA modification information has recently been summarized in RMBase (http://rna.sysu.edu.cn/rmbase/) [62]. Hence, despite RNA modifications in chicken are scarcely studied, the possibility exists that RNA methyltransferase may be highly active in chicken blastodermal cells and leads to 2’-O methylation on rRNA and tRNA, including potentially 3′-end methylation. Alternatively, cleavage of rRNA and tRNA, may also expose 2’-O methylation site at 3′-end, and are therefore included in our 3′-end enriched small RNA population. Further studies may reveal their potential roles in early chicken embryo and germ cell development.

Although our high throughput datasets were generated from multiple chicken breeds, the conclusion of stage-specific piRNA modulation should not be solely attributed to genetic background mediated bias. When taking a full set of piRNA sequencing datasets from blastodermal cells (BC1, BC2), E11 (E11G2) and E14 (E14G2) and adult testes (Adult) sequencing results all from Leghorn breed, we confirmed that all of the stage dependent differences we demonstrated in this paper were also observed within the LH breed (Figs. 1a, c, 2a-c, 4c and Additional file 1: Figure S7B). Studies of genomic occupancy profiling such as ChIP-seq and bisulfite genomic sequencing may further elucidate the links between the PIWI/piRNA pathway and epigenetic regulation along chicken germ cell developmental states. The developmental stage-dependent modulation of TE subfamilies and their importance in establishing or maintaining pluripotency have only recently been appreciated [63–65].

### Chicken PIWI/piRNA pathway machineries may exhibit multiple regulatory strategies

According to the distribution of piRNA counts within targeted genes, PIWI/piRNA might regulate their expressions through various mechanisms. In mice, PIWI/piRNAs regulate TE expression through the ping-pong cycle, which amplifies the piRNAs that lead to the rapid post-transcriptional degradation of TE transcripts and/or facilitate histone modification and de novo methylation for repressing TE transcriptions [12, 32, 58, 66]. The recently discovered phased primary piRNA processing mechanism, which is triggered by initial piRNA targeting, can generate a series of downstream, non-overlapping primary piRNAs through the PIWI slicer activity and therefore enrich piRNA pools [67, 68]. Mouse PiRNAs were also reported to be involved in silencing of protein-coding genes via transcript deadenylation or direct transcript targeting, endonucleolitic cleavage and degradation [69–72]. Nevertheless, while piRNAs have a global repressive effect on TEs, piRNA-mediated post-transcriptional regulation may apply to only a small subset of genes [40]. In support of this notion, the moderate expression changes in the piRNA-associated genes, including *EDIL3* and *PIP5K1B*, before and after spermatogonia formation imply minor roles of the PIWI/piRNA pathway in regulating these genes. Interestingly, however, piRNAs mapped to *EDIL3* showed strong ping-pong cycle features and were not restricted specifically to the embedded TEs (Fig. 3; Additional file 1: Figure S6). In contrast, *PIP5K1B* regions produce piRNA candidates mostly from the sense strand centralized around the embedded TEs, whereas few piRNAs mapped to the antisense strand (Additional file 1: Figure S6). This phenomenon resembles the footprint from the piRNA phasing mechanism [73]. Further analysis of the uniquely mapped piRNAs revealed the possibility that a small number of *trans*-acting antisense piRNAs may trigger the piRNA production from the sense-paired transcript via the phasing mechanism (Additional file 1: Figure S6E-H).

### Stage-dependent regulation over piRNA clusters may evolutionarily acquire roles in germ cell development

We observed that more than half of the overall piRNA clusters displayed strong correlations with the germ cell developmental stages. PiRNA profiling analysis in embryonic gonads allowed the discovery of novel conservation of piRNA clusters between chicken and mammalian species. This finding suggests that the earlier conclusion of the lack of conservation of piRNA clusters between eutherian and chicken was mainly due to a lack of information from the prenatal stages [56]. The conservation of piRNA clusters may be stronger than expected, given the stage-dependency features. Apart from the stage-dependent modulation of TE expressions, our data also implied that the piRNAs produced from E11 and E14 gonadal-enriched piRNA clusters may have preferential targeting to genes involved in neural lineage development and functions. The significant drop of expressions of the piRNA-targeted genes in the E14G germ cell-enriched population suggested potential incidence of repressive roles of chicken piRNAs. The delayed response for piRNA targeting and gene suppression may be partially explained by the involvement of piRNA-guided chromatin repression machinery [34, 74].

The potential roles of developmentally enriched piRNA in repressing neural lineage genes may imply a strengthening germ cell fate during a critical period of spermatogonia stem cell formation. When looking closely into the antisense-mapped piRNAs within these genes, we further observed a stronger reciprocal association between the quantity of these piRNAs and the transcripts bearing intragenic TEs of the same direction (Additional file 4: Table S5). It is therefore possible that while piRNAs modulate the intragenic TE components, they also repress the genes bearing identical sequences. This is reminiscent of the recent finding describing suicide shRNAs that repress genes via the off-target mechanism because of sequence similarity [75]. Our ongoing functional analysis would provide further evidence of how selected genes are specifically targeted by stage-dependent piRNAs while the other genes associated with piRNAs may not receive biologically significant repression. Previous researches have reported that spermatogonial stem cells (SSCs) may regain pluripotency in response to inductive culture condition and adapt a different cell fate including neuron-like cells [76–81]. This suggests that repression of these genes in SSCs is likely reversible. Nevertheless, whether the PIWI/piRNA pathway activities in chicken gonocytes contribute to the establishment of such reversible epigenetic states remains elusive. This will be worth investigating with respect to piRNA-mediated histone modification (H3K9me3) and DNA methylation profiling.

## Conclusions

In this report, we investigated the changes in piRNA compositions in different chicken germ cell developmental stages and explored potential roles of PIWI/piRNA pathways in modulating different stages of germ cell development. We showed a progressive transition of piRNA compositions from TE to intergenic association. Additionally, we showed that piRNAs may be involved in modulating stage-dependent TE expression. Our investigation of the stage-dependent activities of piRNA clusters implied stage-dependent roles in modulating germline development. Unexpectedly, we revealed the potential evolutionary conservation of piRNA clusters between chickens and eutherian mammals.

## Methods

### Animals and tissue sample collection

The fertilized eggs of Cobb500 broiler (*Gallus gallus*) and Leghorn chickens were provided by Taiwan Chunky G.P. Farm Co. and Animal Health Research Institute, Council of Agriculture, Executive Yuan, Taiwan, respectively. Fertilized eggs were incubated at 37.5 °C under 50–60% relative humidity. The protocol has been reviewed and approved by the Institutional Animal Care and Use Committee at National Taiwan University (NTU-100-EL-55; NTU-101-EL-116). For high-throughput small RNA sequencing, samples E11G1 and E14G1 were collected from eggs of the Cobb500 broiler, samples BC1, BC2, E11G2, and E14G2 were collected from eggs of Leghorn layer. For strand-specific mRNA sequencing and quantitative RT-PCR, samples were collected from eggs of JA57 broiler and Leghorn chicken, respectively. Blastoderms were collected from non-incubated eggs. PGCs were isolated from E3 and E7 embryos of the Arbor Acres broiler (*Gallus gallus*) purchased from Chu Lin Farm Co., Ltd., Taiwan. Male gonads were dissected from chicken embryos incubated ranging from 8 days (E8, HH34) to 14 days (E14, HH40) for germ cell isolation. Samples were processed for total and small RNA isolation by TRIzol reagent (Thermo Fisher Scientific, Waltham, MA, USA) and miRNeasy Mini Kit (Qiagen, Valencia, CA, USA), respectively, according to the manufacturers’ instructions. A full set of data solely from Leghorn layer has been extracted from blastodermal cells (BC1, BC2), E11 (E11G2) and E14 (E14G2) and adult testes (Adult) sequencing results to confirm the conclusion.

### High-throughput next-generation sequencing of 3′-end-2’O-methylated small RNAs

RNA extracted from 10 to 15 blastodermal embryos, cultured E7 PGC, 8–10 E11 left-side embryonic gonads, and 8–10 E14 left-side embryonic gonads were preceded for small RNA-sequencing and strand-specific total RNA-sequencing (ssRNA-seq). The quality and concentration of RNA samples were determined using Agilent 2100 Bioanalyzer (Agilent Technologies, Santa Clara, CA, USA). The RNA integrity numbers (RIN) of all our total RNA samples were between 9.9 and 10.0, which indicates minimized RNA degradation induced complication. Small RNA-seq libraries were constructed using the Illumina TruSeq Small RNA sample preparation kit (Illumina, San Diego, CA, USA). For piRNA enrichment, small RNA samples were oxidized by NaIO_4_ before library construction as described (Additional file 1: Figure S1A) [82]. We used the same oxidation treatment protocol that was applied by Li et al. for the adult testes sample preparation [49]. After adaptor ligation, the small RNA libraries were reverse transcribed, followed by amplification through 15 PCR cycles. Sequencing was performed using either an Illumina MiSeq (for E11G1 and E14G1) or an Illumina Solexa Platform (Illumina, San Diego, CA, USA, for BC1, BC2, E7PGC1, E7PGC2, E11G2, E14G2), with read length setting of 50 bp and 75 bp, respectively. Details for ssRNA-seq analysis are described below.

### Bioinformatic filtering for piRNA candidates

Periodate oxidation-treated small RNA-seq datasets for blastodermal cells, E7 PGCs, E11 and E14 gonads were generated in our laboratory. We also obtained the oxidized small RNA sequencing for adult testes (GSM1096613) from Li et al. [49]. For all in-house generated small RNA-seq, we applied adaptor trimming via cutadapt [83] with a 3′ adaptor sequence “TGGAATTCTCGGGTGCCAAGGAACTCCAGTCAC” and parameters -m 15 -M 45 to restrict a small RNA size for the follow-up analysis. We applied FastQC [84] to identify adaptor sequences to be trimmed by cutadapt for adult testes small RNA-seq. Adaptor-trimmed small RNA reads were mapped to the galGal5 genome via Tophat2 (v2.0.12) [85], with parameters -g 1 -N 0 --read-gap-length 0 --read-edit-dist 0 --read-realign-edit-dist 0 --max-insertion-length 0 --max-deletion-length 0, to filter out non-chicken reads and reads containing indels or mismatches (Additional file 1: Table S1). We used chicken galGal5 Refseq transcriptome obtained from UCSC genome browser [86, 87] as the splice junction database to identify reads spanning splice junctions. For stringent piRNA candidate selection, we removed reads mapping to galGal5 rRNA and tRNA sequences from Rfam [88] and Ensembl database (release 87) [89], then to chicken miRNA precursors from miRBase (release 19) [90] and predicted novel miRNA precursors identified via miRDeep (v2.0.0.7; default parameter setting), with parameters -n -c -j -l 18 -m -p [91]. We applied Bowtie (v 1.1.1) [92] with parameters allowing no mismatches for identifying miRNAs with complete sequence match. Finally, we reckoned sequencing reads with 24–34 nt as piRNA candidates (Additional file 1: Figure S1B).

### Strand-specific RNA-seq analysis

We constructed strand-specific paired-end mRNA sequencing for blastodermal cells and E11 and E14 gonads following Illumina TruSeq stranded total RNA sample preparation with 12 PCR-cycle amplification, and then sequenced via Illumina HiSeq sequencing system, with read length setting to 100 bp. The raw RNA-seq reads were curated by trimming adaptors and low score reads, followed by genomic mapping using Tophat2 (v2.0.12) [85] with standard settings for paired-end sequencing. We applied reference guided sequence assembly using Cufflinks (v2.2.1) [93, 94] with default parameters, followed by merging transcriptome assemblies and filtering out potential isoforms. We then annotated the merged contigs based on galGal5 Refseq and Ensembl databases (release 90). Gene expression quantification for each dataset was calculated based on the merged transcriptome annotations, including novel transcripts.

### piRNA cluster analysis

We adapted proTRAC (v2.0.5) and reconfigured it to allow the discovery of dual-stranded piRNA clusters, in addition to single- and bi-directional piRNA clusters [95]. For cross-stage comparison, we merged the piRNA clusters in each profile based on their genomic position. Considering that the transcriptional activities between an expressing piRNA precursor and its sense-overlapping expressing mRNA precursor may not be distinguishable by sequencing information, because both precursors are transcribed via RNA Pol II [96], we extended a candidate piRNA cluster to cover its overlapping transcripts with FPKM > = 1 in at least one in-house RNA-seq dataset (Additional file 1: Figure S8). The expressions of piRNA clusters were measured as the number of piRNA reads per kilobase per million reads (piRPKM) for cross-sample comparison. Data analyses including multidimensional scaling, Heatmap [97], and violin plots [98] were conducted via R [99].

### PGC isolation and in vitro culture

Circulating PGCs (cPGCs) were derived from embryonic blood of E3 embryo (HH15–16), initiated by seeding approximately 2 μL of blood in 48-well plates with 300 μL of FAcs medium [100]. Gonadal PGCs (gPGCs) were derived from E7 embryonic gonads (HH28–30), initiated by seeding homogenized embryonic gonads in 500 μL of FAcs medium in 24-well plates as described [100]. Every 2 days, one-third of the total medium was changed with fresh medium until reaching > 70% confluence. Suspending cells were collected and subcultured in larger wells. One million cells were obtained by in vitro PGC cultures for approximately 1 month for both cPGCs and gPGCs, which were subjected to further RNA isolation processing. PGC cultures were maintained at 37.5 °C incubator with 5% CO_2_ supply.

### Germ cell purification from E11 and E14 embryonic gonads

We harvested male gonads from E11 and E14 embryos. For one batch of purification of the same stage, we pooled approximately 40 E11 embryonic male gonads or approximately 30 E14 embryonic male gonads. The pooled gonads of the same stage were homogenized by Gibco™ 0.25% trypsin-EDTA (Thermo Fisher Scientific, Waltham, MA, USA) treatment at 37 °C for 6 min. The homogenized cells were filtered through 70 μm Falcon™ Cell Strainers (Thermo Fisher Scientific, Waltham, MA, USA); resuspended in DMEM with 1% FBS and 1 mM EDTA supplement at approximately 6 × 10^6^ cells per 150 mm tissue culture treated dish; and then placed in an incubator (37.5 °C, 5% CO_2_) for 4 h. Germ cell-enriched populations (suspension) and germ-depleted populations (adherent) were collected separately and dissolved in TRIzol reagent (Thermo Fisher Scientific, Waltham, MA, USA) for RNA extraction. We kept a small portion of the sample to check for germ cell enrichment efficiency via immunofluorescent staining.

### Immunocytochemistry

We applied immunofluorescent staining to evaluate the germ cell enrichment efficiency. Cells were loaded onto slides, fixed (4% PFA in PBS) for 20 min at room temperature, quenched (10 mM Tris pH 7.5, 50 mM KCl, 20 mM EDTA) for 5 min, permeabilized (0.1% Saponin in PBS) for 5 min, with PBS wash in each step. Samples were blocked (1% BSA in PBS) overnight at 4 °C. The cells were incubated at room temperature with primary anti-CVH antibody (#9C4-2E4; Biotem, Apprieu, France) at 1:1000 dilution in 1% BSA for 1 h, washed several times with PBS, and then incubated with secondary goat anti-mouse DyLight® 594 antibody (#ab96873; Abcam, Cambridge, MA, USA) at 1:10000 dilution in 1% BSA for 1 h. We then applied Hoechst 33,342 nucleic acid staining (Thermo Fisher Scientific, Waltham, MA, USA) at 1:10000 dilution in 1% BSA for 5 min. After several washes, samples were mounted and stored at − 20 °C. Fluorescent images were observed under a fluorescence microscope. Germ cell-enriched populations with isolation efficiency above 80% were used for the subsequent expression analysis via RT-qPCR.

### RT-qPCR primer design

All in-house developed primers were designed via Primer3 [101, 102] against gene or transcript sequences obtained from UCSC genome browser [86, 87]. For the design of primers against chicken LINE sequences, we obtained 10 longest sequences for each of CR1-B to CR1-H from the galGal5 genome sequence, and then identified consensus sequences via R-packages [99] for Multiple Sequence Alignment (msa) [103] and Biological Sequences Retrieval and Analysis (seqinr) [104]. Primer target specificity was validated via in-silico PCR in UCSC genome browser [87].

### Total RNA isolation from enriched germ cells and reverse transcription real-time PCR (RT-qPCR)

For total RNA extraction, equal volume of ethanol was added to a sample in TRIzol, and then loaded onto a filter column provided by the miRNeasy Mini Kit (Qiagen, Valencia, CA, USA). The RNA extraction protocol was then performed according to the manufacturers’ instruction, with the exception of two-time DNase treatments (once on-column, and once prior to cDNA synthesis) to ensure minimal DNA contamination. One microgram (1 μg) of extracted RNAs was reverse transcribed according to instructions provided by the Invitrogen™ SuperScript® III First-Strand Synthesis System (Thermo Fisher Scientific, Waltham, MA, USA). The temperature settings for reverse transcription were 5 min at 25 °C, 60 min at 50 °C, and 15 min at 70 °C, and the samples were then chilled on ice. For qPCR, KAPA SYBR® FAST qPCR Master Mix (Kapa Biosystems, Wilmington, MA, USA) was used, followed by real-time PCR in Roche Light Cycler® 480II for 10 min 95 °C preheat, then 40 PCR cycles of 10 s at 95 °C, 10 s at 50 °C, and 10 s at 70 °C. The primers used for qPCR are listed in Additional file 1: Table S6.

## Additional files

Additional file 1: **Figure S1.** High-confidence piRNA candidate identification pipeline. **Figure S2.** Examination of germ cell isolation efficiency using immunocytochemistry. **Figure S3.** Expression of potential spermatogonial stem cell (SSC) markers and genes encoding PIWI/piRNA pathway components in embryonic germ cells. **Figure S4.** Scheme for assessing ping-pong cycle piRNAs. **Figure S5.** Genomic association for piRNA candidates at each stage at a specific piRNA length. **Figure S6.** PiRNA distribution at the associated transcripts that reciprocally expressed compare to the quantity of mapped piRNAs (EDIL3, ANGPTL2, and PIP5K1B). **Figure S7.** PiRNA cluster analysis results. **Figure S8.** Schematic presentation of merging piRNAs from multiple stages. **Figure S9.** Venn diagram of number of transposable elements (TEs) and genes targeted by piRNAs from stage-enriched piRNA clusters. **Figure S10.** Stage-enriched piRNA clusters may contribute to stage-dependent regulatory roles. **Figure S11.** Ontology analysis of gene sets targeted by piRNAs from stage-enriched piRNA clusters under Enrichr: GO Biological Process 2015 category. **Figure S12.** Expression analysis via RT-qPCR on piRNA targeted genes associated with neural development. **Table S1.** Bioinformatic filtering results from high-confidence piRNA identification pipeline. **Table S2.** Number of piRNAs (in piRPM) mapped to TEs embedded in the transcripts that are differentially associated between E11G and E14G piRNAs. **Table S3.** Number of clusterable piRNAs before and piRNA cluster boundaries after adjustment. **Table S4.** Genes and TEs targeted by stage-enriched piRNA cluster-derived piRNAs. **Table S5.** Number of piRNAs (in piRPM) mapped to TEs embedded in the transcripts that are highly associated with piRNAs enriched in embryonic (E11 and E14) gonadal piRNA clusters (EG-piRC). **Table S6.** RT-qPCR primer sets. (DOCX 3433 kb)

Additional file 2: **Table S2.** Number of piRNAs (in piRPM) mapped to TEs embedded in the transcripts that are differentially associated between E11G and E14G piRNAs. (XLS 109 kb)

Additional file 3: **Table S4.** Genes and TEs targeted by stage-enriched piRNA cluster-derived piRNAs. (XLS 69 kb)

Additional file 4: **Table S5.** Number of piRNAs (in piRPM) mapped to TEs embedded in the transcripts that are highly associated with piRNAs enriched in embryonic (E11 and E14) gonadal piRNA clusters (EG-piRC). (XLS 74 kb)

## Abbreviations

1U: Enriched uracil at the 1st nucleotide
10A: Enriched adenine at the 10th nucleotide
AT: Adult testes
AT-piRISC: Adult testes-enriched piRNA cluster
BC: Blastodermal cell
BC-piRC: BC-enriched piRNA cluster
CR1: Chicken repeat 1 element
E11G: Embryonic day 11 gonads
E14G: Embryonic day 14 gonads
E7PGC: Cultured PGCs from E7 gonads
EG-piRC: E11 and E14 gonadal-enriched piRNA cluster
GO: Gene ontology
H3K9me3: Tri-methylation on histone H3 lysine 9
HH: Hamburger–hamilton stages
LINE: Long interspersed nuclear element
LTR: Long terminal repeats
PCR: Polymerase chain reaction
PGC: Primordial germ cell
PGC-piRC: PGC-enriched piRNA cluster
piRISC: piRNA induced silencing complex
PTGS: Post-transcriptional gene silencing
SSC: Spermatogonial stem cell
TE: Transposable element
TGS: Transcriptional gene silencing
