# Correction to: Stage-dependent piRNAs in chicken implicated roles in modulating male germ cell development

**DOI:** 10.1186/s12864-018-4863-y

**Published:** 2018-06-19

**Authors:** Kai-Wei Chang, Yen-Tzu Tseng, Yi-Chen Chen, Chih-Yun Yu, Hung-Fu Liao, Yi-Chun Chen, Yu-Fan Evan Tu, Shinn-Chih Wu, I-Hsuan Liu, Marina Pinskaya, Antonin Morillon, Bertrand Pain, Shau-Ping Lin

**Affiliations:** 1 Genome and Systems Biology Degree Program, National Taiwan University, Taipei, 106 Taiwan; 2 Institute of Biotechnology, National Taiwan University, Taipei, 106 Taiwan; 3 Department of Animal Science and Technology, National Taiwan University, Taipei, 106 Taiwan; 4 ncRNA, epigenetic and genome fluidity, Institut Curie, Centre de Recherche, CNRS UMR 3244, PSL Research University, Université Pierre et Marie Curie, F-75005 Paris, France; 5 Univ Lyon, Université Lyon 1, INSERM, INRA, Stem Cell and Brain Research Institute, U1208, USC1361, F-69500 Bron, France; 6 Research Center for Developmental Biology and Regenerative Medicine, National Taiwan University, Taipei, 106 Taiwan; 7 Agricultural Biotechnology Research Centre, Academia Sinica, Taipei, 106 Taiwan; 8 Center for Systems Biology, National Taiwan University, Taipei, 106 Taiwan; 9 Present Address: Graduate Institute of Brain and Mind Sciences, College of Medicine, National Taiwan University, Taipei, 10051 Taiwan

## Correction

Following publication of the original article [1], the authors reported that one of the authors’ names is spelled incorrectly. In this Correction the incorrect and correct author name are shown. The original publication of this article has been corrected.

Originally the author name was published as:

- Antonin Morillion

**The correct author name is:**

- Antonin Morillon
