# Supplementary material for: Stage-dependent piRNAs in chicken implicated roles in modulating male germ cell development
Source: BMC Genomics. 2018 Jun 1;19:425. doi: 10.1186/s12864-018-4820-9 (PMC5984780; doi:10.1186/s12864-018-4820-9)
Supplement: Supplementary file 1 — Figure S1. High-confidence piRNA candidate identification pipeline. Figure S2. Examination of germ cell isolation efficiency using immunocytochemistry. Figure S3. Expression of potential spermatogonial stem cell (SSC) markers and genes encoding PIWI/piRNA pathway components in embryonic germ cells. Figure S4. Scheme for assessing ping-pong cycle piRNAs. Figure S5. Genomic association for piRNA candidates at each stage at a specific piRNA length. Figure S6. PiRNA distribution at the associated transcripts that reciprocally expressed compare to the quantity of mapped piRNAs (EDIL3, ANGPTL2, and PIP5K1B). Figure S7. PiRNA cluster analysis results. Figure S8. Schematic presentation of merging piRNAs from multiple stages. Figure S9. Venn diagram of number of transposable elements (TEs) and genes targeted by piRNAs from stage-enriched piRNA clusters. Figure S10. Stage-enriched piRNA clusters may contribute to stage-dependent regulatory roles. Figure S11. Ontology analysis of gene sets targeted by piRNAs from stage-enriched piRNA clusters under Enrichr: GO Biological Process 2015 category. Figure S12. Expression analysis via RT-qPCR on piRNA targeted genes associated with neural development. Table S1. Bioinformatic filtering results from high-confidence piRNA identification pipeline. Table S2. Number of piRNAs (in piRPM) mapped to TEs embedded in the transcripts that are differentially associated between E11G and E14G piRNAs. Table S3. Number of clusterable piRNAs before and piRNA cluster boundaries after adjustment. Table S4. Genes and TEs targeted by stage-enriched piRNA cluster-derived piRNAs. Table S5. Number of piRNAs (in piRPM) mapped to TEs embedded in the transcripts that are highly associated with piRNAs enriched in embryonic (E11 and E14) gonadal piRNA clusters (EG-piRC). Table S6. RT-qPCR primer sets. (DOCX 3433 kb) [file 12864_2018_4820_MOESM1_ESM.docx]

[Figure S1. High-confidence piRNA candidate identification pipeline. 2](#_Toc510963989)

[Figure S2. Examination of germ cell isolation efficiency using immunocytochemistry. 3](#_Toc510963990)

[Figure S3. Expression of potential spermatogonial stem cell (SSC) markers and genes encoding PIWI/piRNA pathway components in embryonic germ cells. 4](#_Toc510963991)

[Figure S4. Scheme for assessing ping-pong cycle piRNAs. 5](#_Toc510963992)

[Figure S5. Genomic association for piRNA candidates at each stage at a specific piRNA length. 6](#_Toc510963993)

[Figure S6. PiRNA distribution at the associated transcripts that reciprocally expressed compare to the quantity of mapped piRNAs (EDIL3, ANGPTL2, and PIP5K1B). 7](#_Toc510963994)

[Figure S7. PiRNA cluster analysis results. 8](#_Toc510963995)

[Figure S8. Schematic presentation of merging piRNAs from multiple stages. 9](#_Toc510963996)

[Figure S9. Venn diagram of number of transposable elements (TEs) and genes targeted by piRNAs from stage-enriched piRNA clusters. 10](#_Toc510963997)

[Figure S10. Stage-enriched piRNA clusters may contribute to stage-dependent regulatory roles. 11](#_Toc510963998)

[Figure S11. Ontology analysis of gene sets targeted by piRNAs from stage-enriched piRNA clusters under Enrichr: GO Biological Process 2015 category. 12](#_Toc510963999)

[Figure S12. Expression analysis via RT-qPCR on piRNA targeted genes associated with neural development. 13](#_Toc510964000)

[Table S1. Bioinformatic filtering results from high-confidence piRNA identification pipeline. 14](#_Toc510964001)

[Table S2. Number of piRNAs (in piRPM) mapped to TEs embedded in the transcripts that are differentially associated between E11G and E14G piRNAs. 15](#_Toc510964002)

[Table S3. Number of clusterable piRNAs before and piRNA cluster boundaries after adjustment 16](#_Toc510964003)

[Table S4. Genes and TEs targeted by stage-enriched piRNA cluster-derived piRNAs 17](#_Toc510964004)

[Table S5. Number of piRNAs (in piRPM) mapped to TEs embedded in the transcripts that are highly associated with piRNAs enriched in embryonic (E11 and E14) gonadal piRNA clusters (EG-piRC). 17](#_Toc510964005)

[Table S6. RT-qPCR primer sets 18](#_Toc510964006)

[References 16](#_Toc502695714)

Figure S1. High-confidence piRNA candidate identification pipeline.

**(A)** Oxidation enrichment for 3’-end 2’-O-methylated chicken piRNAs followed by adaptor ligation for small RNA sequencing. **(B)** Bioinformatic pipeline for identifying high-confidence piRNA candidates from oxidation-treated small RNA-seq samples. Reads are hierarchically filtered out rRNA/tRNA, miRNA, and miRDeep2 predicted miRNAs. Reads within 24-34nt are considered high-confidence piRNAs. **(C)** Proportion of chicken small RNA types with and without oxidation enrichment treatment. Germ cell developmental stages included blastodermal cells (BC), E7 primordial germ cells (E7PGC), E11 gonads (E11G), E14 gonads (E14G), and adult testes acquired from GSM1096613.

s
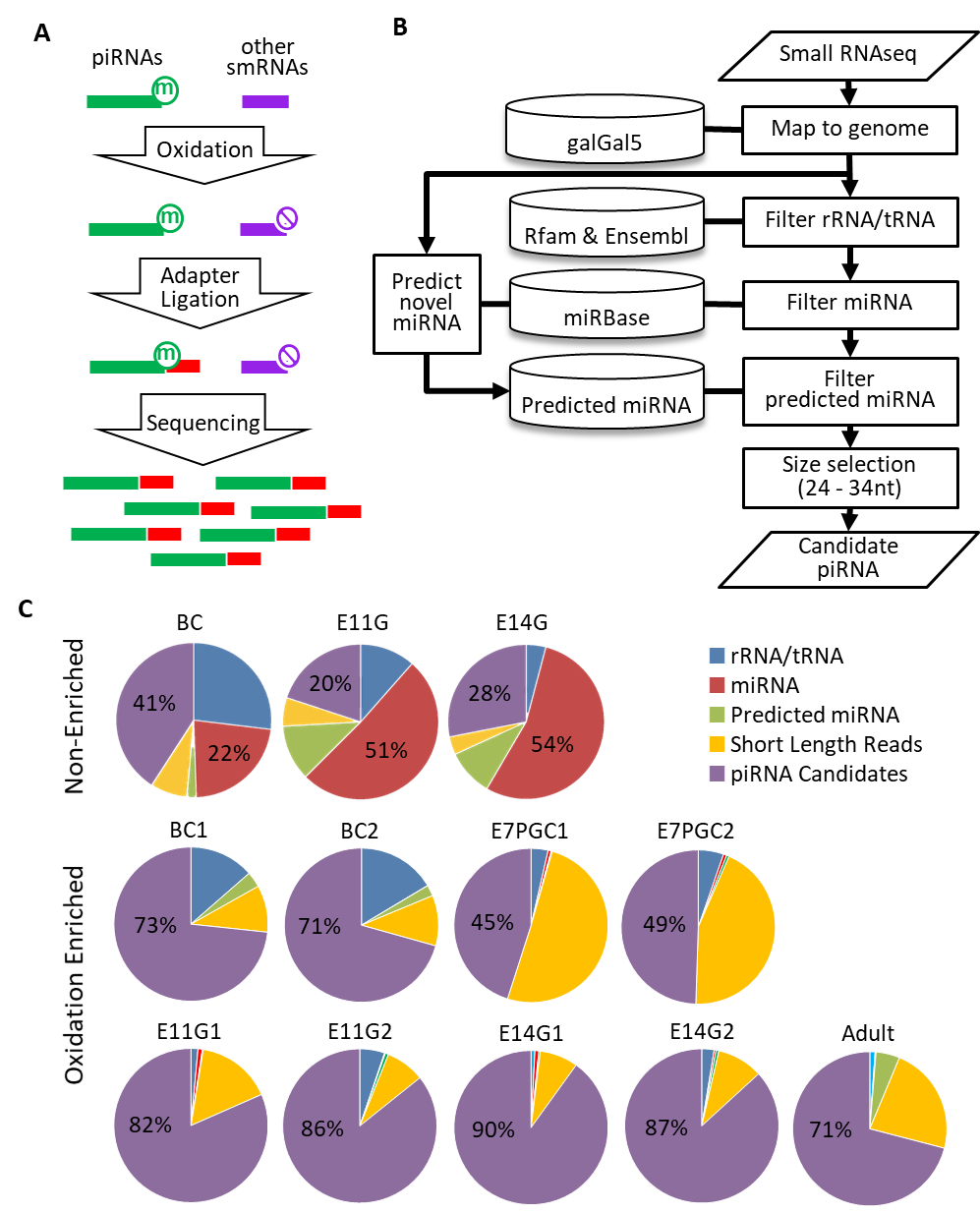


Figure S2. Examination of germ cell isolation efficiency using immunocytochemistry.

Immunocytochemistry staining against chicken germ cell marker CVH (VASA) for evaluating germ cell enrichment efficiency on E11G and E14G. Hoechst 33342 was used for nuclear staining. Germ cell-enriched populations with isolation efficiency above 80% were used for the subsequent expression analysis via RT-qPCR.


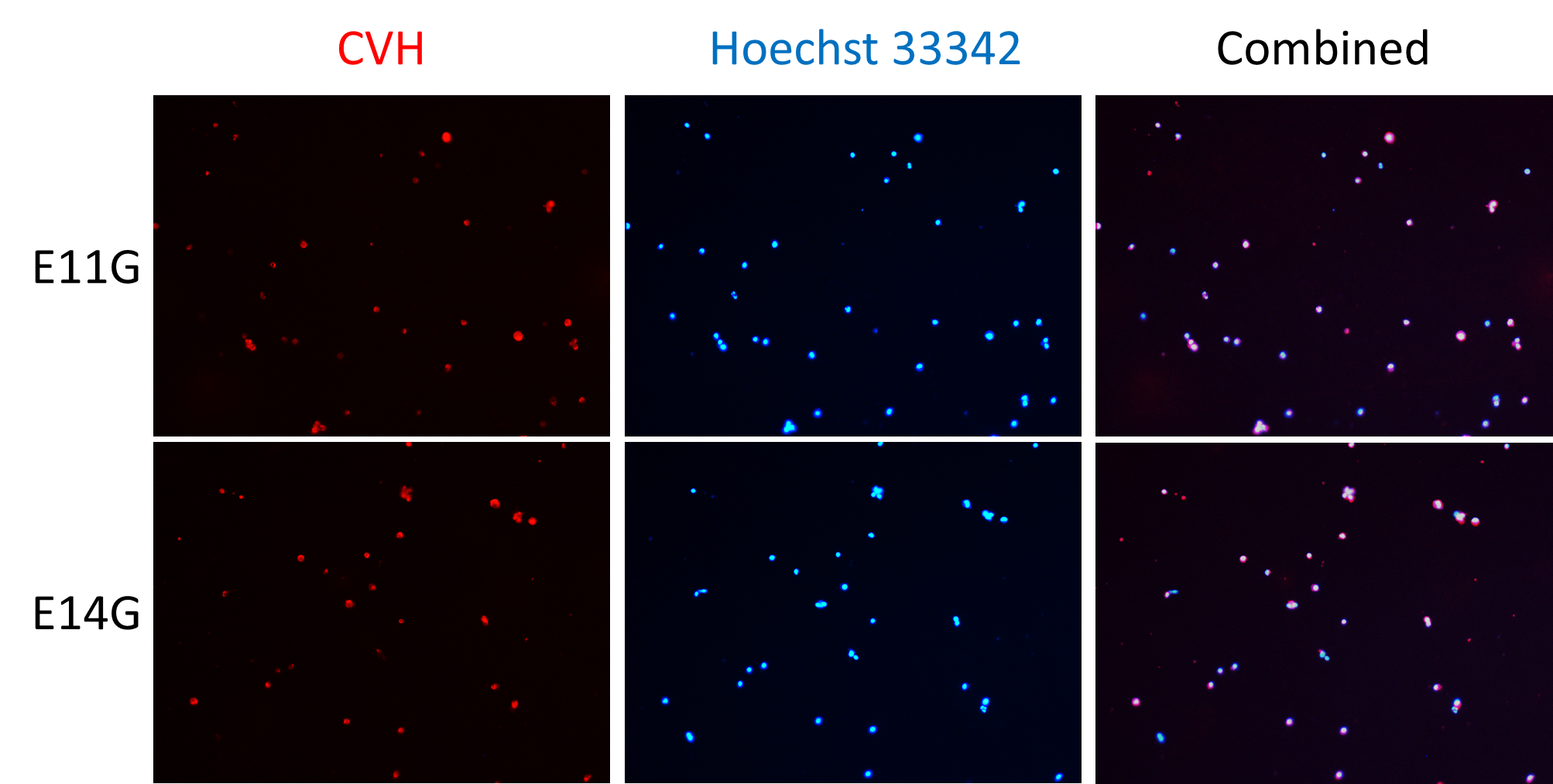


Figure S3. Expression of potential spermatogonial stem cell (SSC) markers and genes encoding PIWI/piRNA pathway components in embryonic germ cells.

Germ cells from E8 to E14 male gonads were freshly enriched for the gene expression analysis. **(A)** Relative expression levels of germ cell-associated genes, and **(B)** PIWI pathway related genes, were examined by quantitative RT-qPCR. Expression of spermatogonia markers, THY1, GFRA1, and DAZL, are detected in enriched male germ cells from embryonic day 8 onwards. Repression of pluripotent marker BLIMP1 and progressive increase of CVH correlated well with advanced male germ cell development. Strong expressions of PIWI/piRNA genes, including PIWIL1 (CIWI), PIWIL2 (CILI), GPAT2, TDRD1, TDRD9, MAEL, and GASZ, supported the active PIWI/piRNA pathway in germ cell-enriched population from E8 to E14 gonads. Each data point represents the average of at least three independent experiments (N>=3), in which 60-80 male gonads were harvested for germ cell isolation for each batch.


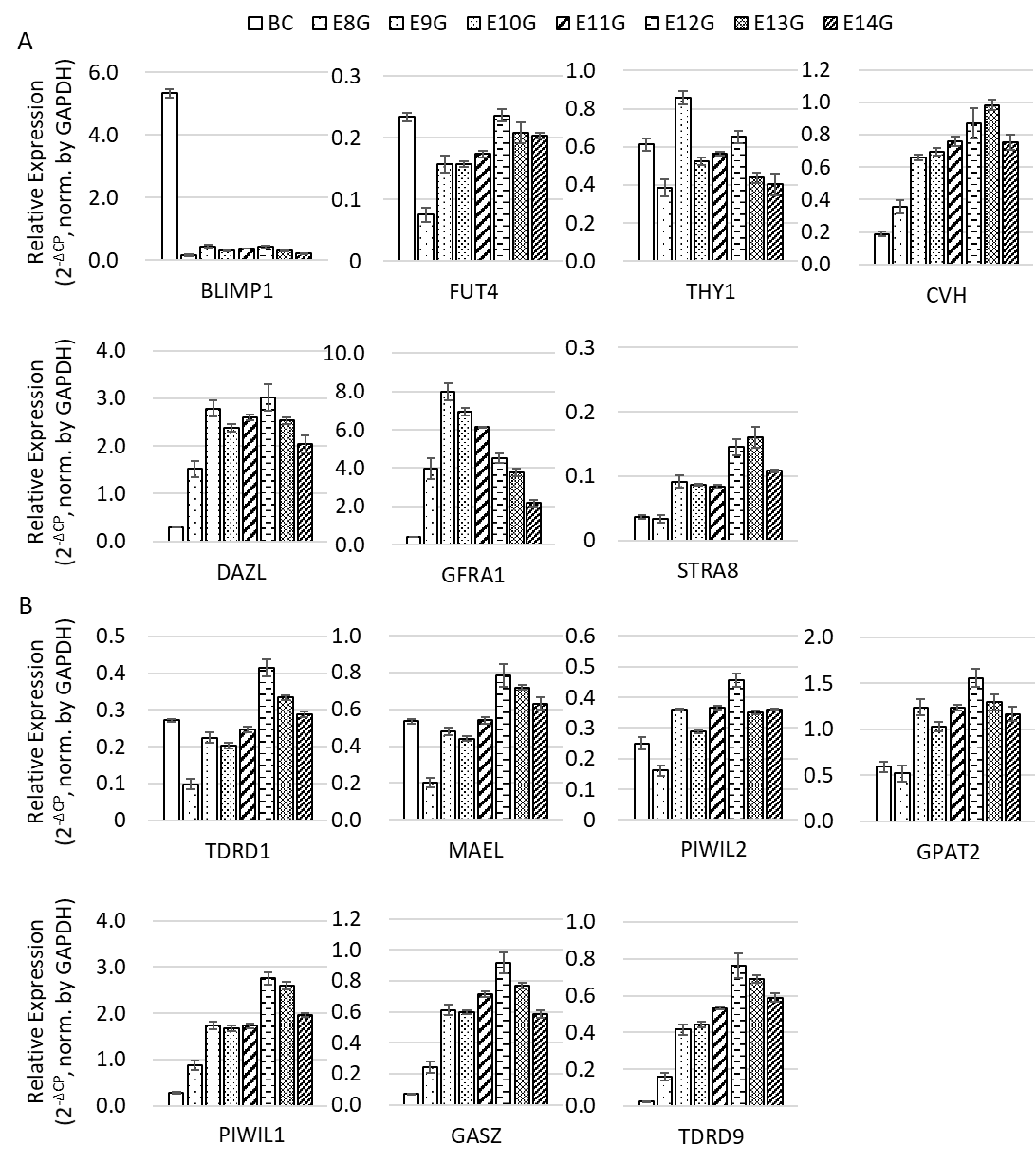


Figure S4. Scheme for assessing ping-pong cycle piRNAs.

PiRNA served as a guiding sequence for targeting in the piRNA-mediated transcriptional silencing, including the ping-pong cycle. Since PIWI mediated cleavage occurs at the 5’ 10 bp region, a pair of ping-pong piRNA reads must be mapped to the same genomic region with 5’ antisense overlapping. We therefore use these features in our bioinformatic pipeline to identify “ping-pong cycle derived piRNAs” for further analysis.


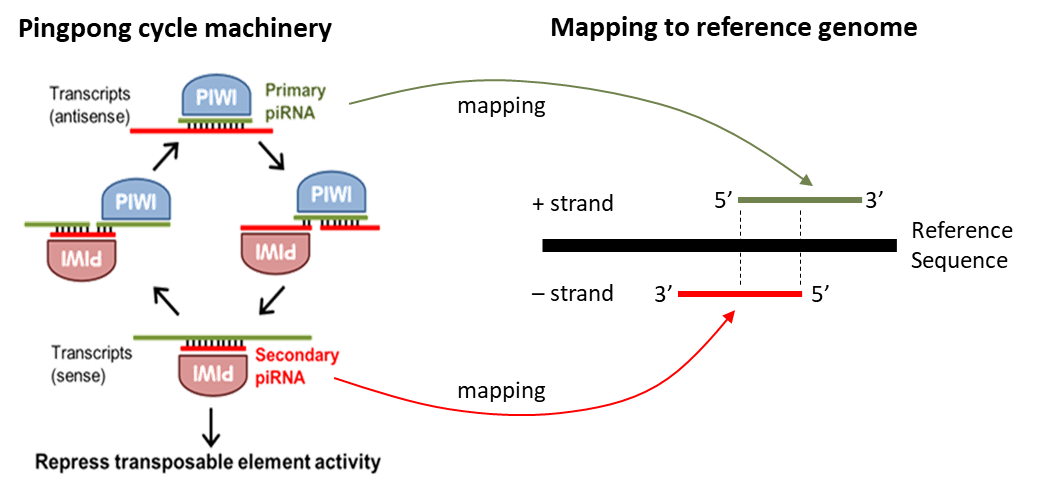


Figure S5. Genomic association for piRNA candidates at each stage at a specific piRNA length.

Genomic association of the shorter (24~25 nt; **A**), or longer (28 nt; **B**) piRNAs from each developmental stage. The shorter and longer piRNAs are particularly enriched in adult testes and blastodermal cells, respectively.


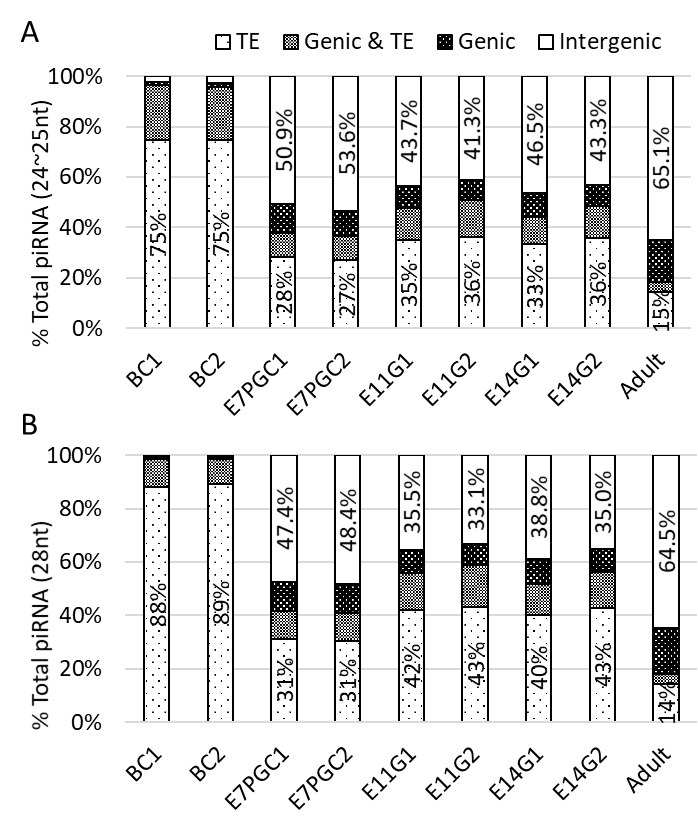


Figure S6. PiRNA distribution at the associated transcripts that reciprocally expressed compare to the quantity of mapped piRNAs (EDIL3, ANGPTL2, and PIP5K1B).

**(A)** PiRNA reads of individual target transcripts were calculated according to genomic location at either exon or intron of the transcripts. **(B)** Proportion of PiRNA reads associated with repeat element (black) and ping-pong cycle signature (grey), in the context of their association with candidate genes of interest. Genomic mapping of piRNAs onto **(C, D)** EDIL3; **(E, F)** ANGPTL2; and **(G, H)** PIP5K1B are shown with respect to E11G and E14G samples. The landscape of piRNA reads of entire (upper panel) and partial transcripts (bottom panel) were shown as bar charts. The vertical axis shows piRNA read counts at the sense and antisense directions according to the respective gene. The sketch map of the relative locations of TEs is indicated by arrows in the bottom of each panel.


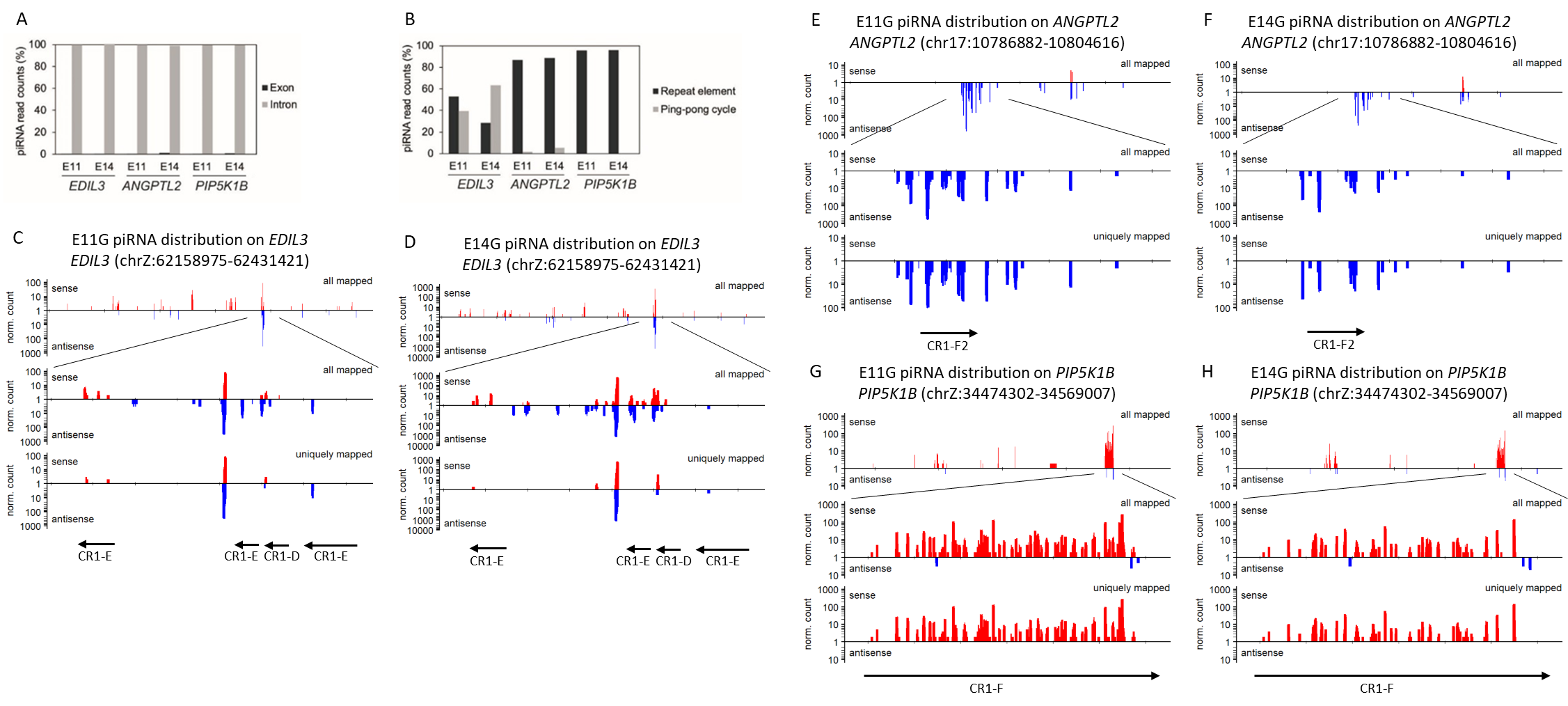


Figure S7. PiRNA cluster analysis results.

**(A)** Length distribution of piRNA clusters, at a minimum length setting of 3 kb, and p-value less than 0.05. PiRNA cluster loci boundaries are adjusted across samples and gonadal RNA-seq transcriptome assembly (see METHOD). The number of piRNA clusters identified in each sample is recorded, as indicated in brackets. A total of 7269 clusters are identified after adjustment. **(B)** Heatmap for piRNA cluster expression in each sample. Expression is calculated by piRNAs per million reads per Kb (piRPKM). The clustering method in Figure S7B is the “Ward’s hierarchical agglomerative clustering method” [1] implemented as ward.D in R.


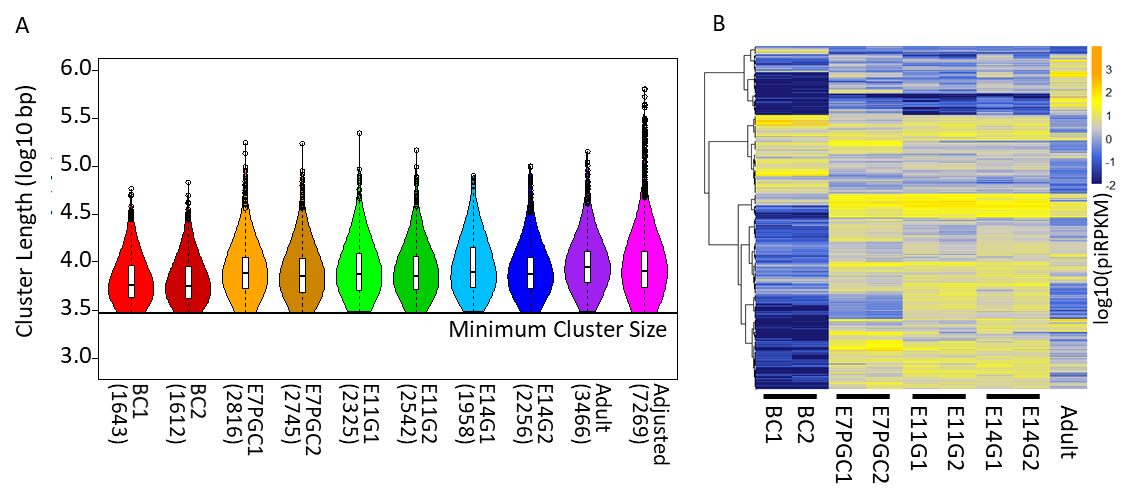


Figure S8. Schematic presentation of merging piRNAs from multiple stages.

PiRNA clusters from different stages are merged if they occupy overlapping genome locations. PiRNA cluster boundaries are adjusted by the overlapping transcripts with FPKM >= 1. This process standardizes piRNA clusters for cross-stage comparison.


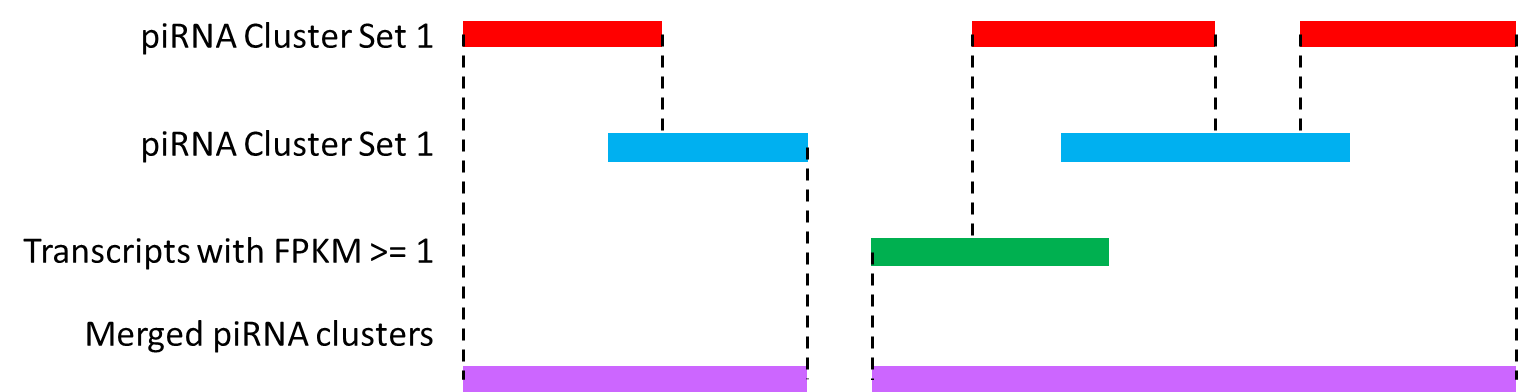


Figure S9. Venn diagram of number of transposable elements (TEs) and genes targeted by piRNAs from stage-enriched piRNA clusters.

**(A)** Among the TE families targeted by piRNAs, most of them were targeted by piRNAs from several developmental stages. The pie chart denotes the proportion of TEs being targeted by piRNAs, where CR1 and ERVL are predominantly targeted. Because TEs are highly repeated sequences occupying multiple genomic loci, TEs targeted by piRNAs by at least 100 piRPM are deemed potentially targeted by piRNAs. **(B)** Hundreds of piRNA associated genes from each developmental stage studied, may be targeted by piRNAs in a stage-dependent manner. Stage-enriched piRNA clusters from blastodermal cells (BC), primordial germ cells (PGC), embryonic gonads from E11 and E14 (EG), and adult testes (AT) are included. Genes targeted by piRNAs by at least 10 piRPM are deemed potentially targeted by piRNAs.


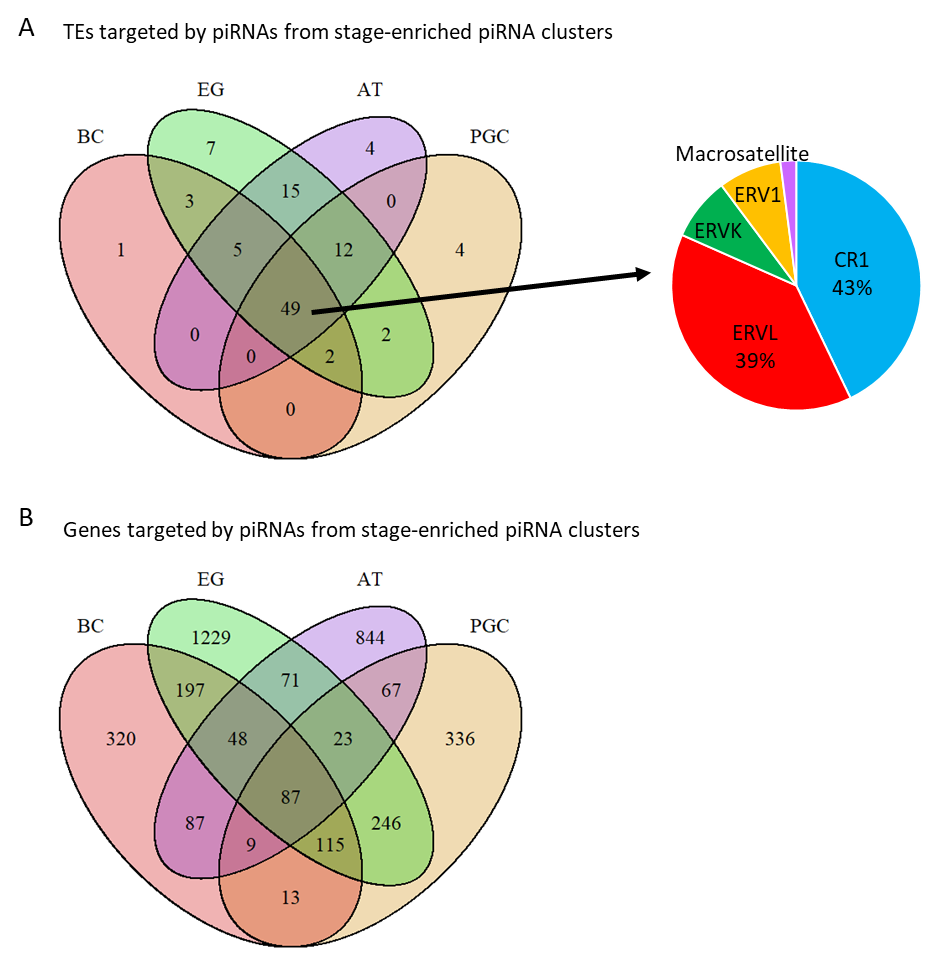


Figure S10. Stage-enriched piRNA clusters may contribute to stage-dependent regulatory roles.

**(A)** Enrichment results against gene sets under Enrichr: Mouse Gene Atlas category. **(B)** Enrichment results against gene sets under Enrichr: GO Biological Process 2015 category, focused on genes targeted by EG-piRC originated piRNA candidates. The Enrichr score is the combined score from the Enrichr website, described as the “multiplication of log of p-value from Fisher exact test and z-score of deviation from expected rank” [2]. The cutoff line represents a p-value = 0.05, high Enrichr score correspond with low p-value. PiRNAs derived from stage-enriched piRNA clusters from blastodermal cells (BC-piRC), primordial germ cells (PGC-piRC), embryonic gonads from E11 and E14 (EG-piRC), and adult testes (AT-piRC) are included.


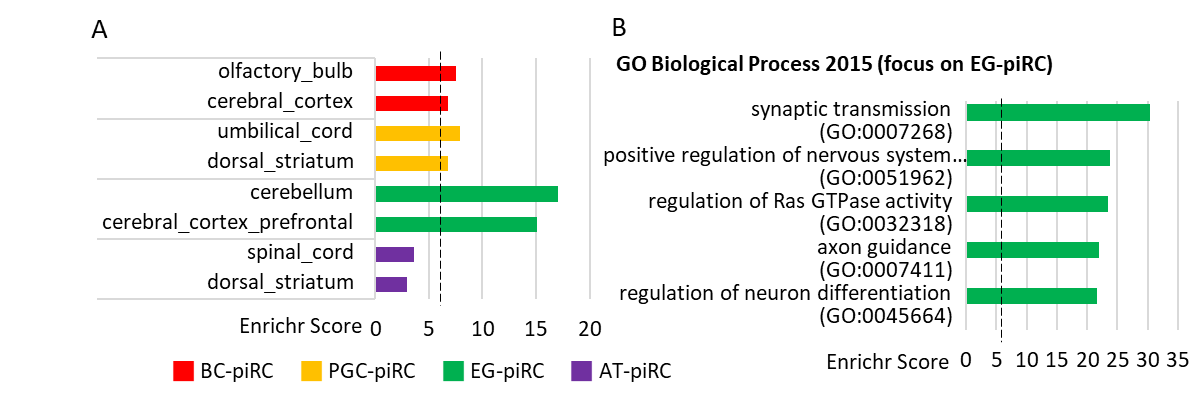


Figure S11. Ontology analysis of gene sets targeted by piRNAs from stage-enriched piRNA clusters under Enrichr: GO Biological Process 2015 category.


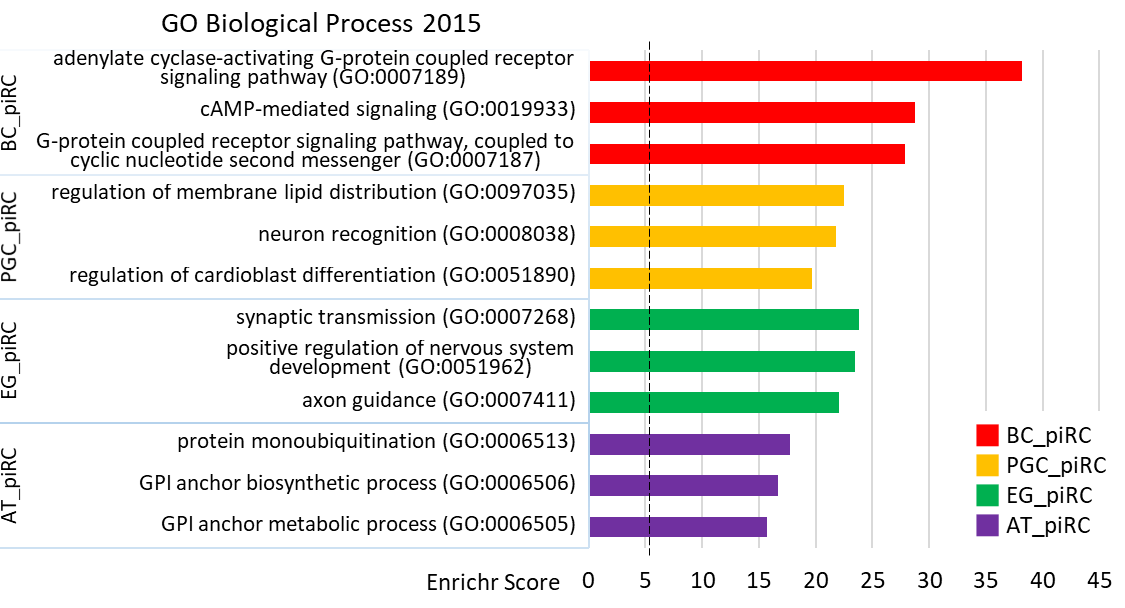


Figure S12. **Expression analysis via RT-qPCR on piRNA targeted genes associated with neural development.**

The tested samples include cultured E3 circulating PGC (E3PGC), E7 gonadal PGC (E7PGC), the germ cell-enriched population from E11 and E14 gonads (E11Germ and E14Germ), and E8 brain. Statistical significance (p<0.05) is denoted as italic *a* – *f*, for pairwise expression comparisons of E3PGC vs E7PGC; E3PGC vs E11Germ; E3PGC vs E14Germ; E7PGC vs E11Germ; E7PGC vs E14Germ; and E11Germ vs E14Germ; respectively. PiRNA expression associated with each listed gene is calculated as piRPM.


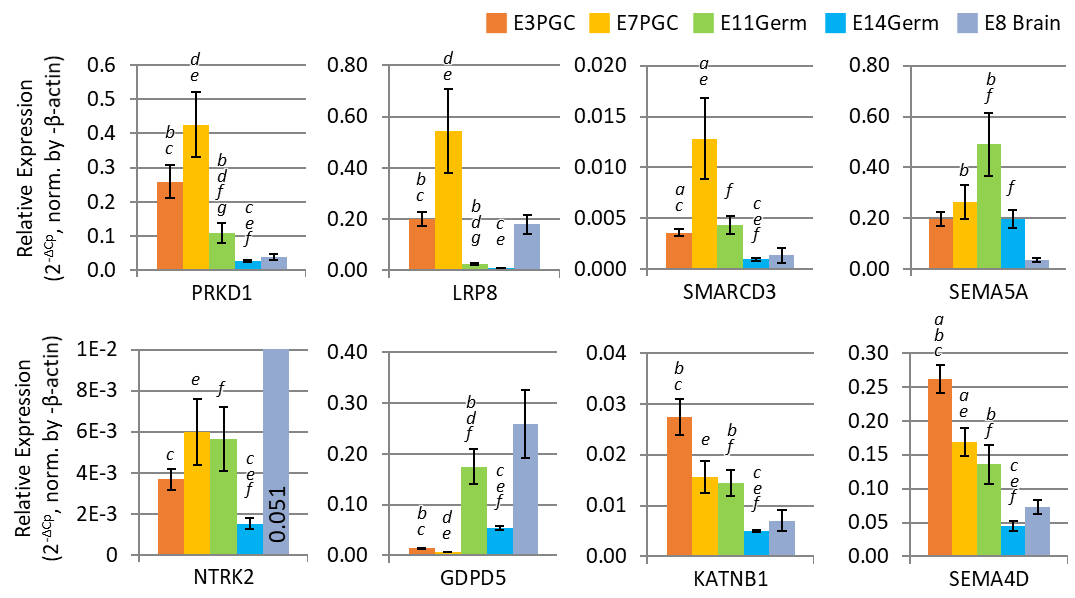


Table S1. Bioinformatic filtering results from high-confidence piRNA identification pipeline.

|  | **BC1** | **BC2** | **E7PGC1** | **E7PGC2** | **E11G1** | **E11G2** | **E14G1** | **E14G2** | **Adult** |
| --- | --- | --- | --- | --- | --- | --- | --- | --- | --- |
| **Input** | 11479145 | 11933742 | 26967088 | 18937359 | 2299878 | 11164889 | 2410462 | 12257693 | 123441738 |
| **Mapped (percent mapped)** | 8641650  (75%) | 9032738  (76%) | 23976074  (89%) | 16038004  (85%) | 1711254  (74%) | 9185728  (82%) | 1843702  (76%) | 10226026  (83%) | 92601907  (75%) |
| **rRNA/tRNA** | 1171244 | 1481592 | 856770 | 855864 | 25080 | 478069 | 14883 | 263457 | 1027955 |
| **miRNA** | 2086 | 3842 | 149788 | 106340 | 15535 | 16500 | 15037 | 41790 | 183338 |
| **predicted miRNA** | 281667 | 207893 | 59547 | 90337 | 3100 | 62600 | 5603 | 59845 | 4617244 |
| **short reads (<24 nt)** | 850072 | 957084 | 12106756 | 7047655 | 271382 | 751741 | 147969 | 985460 | 21068454 |
| **piRNA candidates** | 6336581 | 6382327 | 10803213 | 7937808 | 1396157 | 7876818 | 1660210 | 8875474 | 65704916 |

Table S2. Number of piRNAs (in piRPM) mapped to TEs embedded in the transcripts that are differentially associated between E11G and E14G piRNAs.

PiRPMs mapped to TEs and non-TEs within genes of interests are calculated in Additional File 2: Table S2

Table S3. Number of clusterable piRNAs before and after piRNA cluster boundary adjustment

|  | **Total piRNA** | **Clusterable piRNA**s | | | | |
| --- | --- | --- | --- | --- | --- | --- |
|  |  | Before Adjust | | | After Adjust | |
| **BC1** | 6336581 | 3902290 | (61.58%) | 4328642 | | (68.31%) |
| **BC2** | 6382327 | 3882668 | (60.83%) | 4348720 | | (68.14%) |
| **E7PGC1** | 10803213 | 7002990 | (64.85%) | 8355562 | | (77.37%) |
| **E7PGC2** | 7937808 | 5056815 | (63.73%) | 6279679 | | (79.14%) |
| **E11G1** | 1396157 | 784810 | (56.24%) | 992529 | | (71.13%) |
| **E11G2** | 7876818 | 4692270 | (59.57%) | 5704125 | | (72.42%) |
| **E14G1** | 1660210 | 849878 | (51.22%) | 1139004 | | (68.64%) |
| **E14G2** | 8875474 | 4794055 | (54.01%) | 6205828 | | (69.92%) |
| **Adult** | 65704916 | 52951459 | (80.59%) | 55641689 | | (84.68%) |

Table S4. Genes and TEs targeted by stage-enriched piRNA cluster-derived piRNAs

Gene and TE lists are listed in Additional File 3: Table S4

Table S5. Number of piRNAs (in piRPM) mapped to TEs embedded in the transcripts that are highly associated with piRNAs enriched in embryonic (E11 and E14) gonadal piRNA clusters (EG-piRC).

PiRPMs mapped to TEs and non-TEs within genes of interests are calculated in Additional File 4: Table S5

Table S6. RT-qPCR primer sets

| **Primer set** | **Forward Primer** | **Reverse Primer** | **Accession** | **Reference** |
| --- | --- | --- | --- | --- |
| *CVH* | CCTTGCAGCCTTTCTTTGTC | ACGACCAGTTCGTCCAATTC | NM_204708 | [3] |
| *GAPDH* | GGTGGTGCTAAGCGTGTTAT | ACCTCTGCCATCTCTCCACA | NM_204305 | [4] |
| *CIWI* | CCGAAATGGAGAAGATGTGAGGA | TGTGATTAGGGATGCTGACTGG | NM_001098852 | [4] |
| *CILI* | GCTCCGTCATCGGCTTCGT | TTCTTGGGCAGGCAGTGGTT | JN248386.1 | [4] |
| *THY1* | GAACGTCTACCGGAACCGAG | AGCCACGAGGTGTTCTGGAT | NM_204381.2 | This study |
| *POU5F1* | TGAAGGGAACGCTGGAGAGC | ATGTCACTGGGATGGGCAGAC | NM_001110178 | [3] |
| *PLZF* | TTGGATGACCTGCTCTAC | GGAATGCTTGGAGATGAAG | XM_417898.4 | This study |
| *GFRA1* | AGTCACGCTCTGTTAGTAG | GCATTCATCTATGTCATTACCA | NM_205102.1 | This study |
| *FUT4* | GGACTACATCACCGAGAA | CGAGGAACTTCAGGTAGG | NM_001031487.1 | This study |
| *GPAT2* | TCTCCTTCCTCCTCTTCT | GTCACTCAATGCCTGTTC | ENSGALT00000039021 | This study |
| *TDRD1* | GTCAGCAGATACACATTGG | CTATAACAGCAGCACGATAC | ENSGALT00000014592 | This study |
| *TDRD9* | AAGAGATTCCAAGCAGTCT | AATACACCTCCACAAGAAGA | ENSGALT00000018863 | This study |
| *MAEL* | TTCTTGATGCCTCTGCTAT | CTTGACACACCCGATTTC | ENSGALT00000024921 | This study |
| *GASZ* | ATCCTGAAGACCGTAGAAC | ATGAGCAACAAGAAGACTAAC | NM_001135165.1 | This study |
| *EDIL3* | AGTCCAGAATATGTGAAGT | GAGGAGTGAAGGAGTTAG | ENSGALT00000025199 | This study |
| *ANGPTL2* | ACCTACCTTACCAACCAT | GCCATCTTCTAATGCTTGTA | NM_001277699 | This study |
| *PIP5K1B* | TGAGCCTTGTAGATAGAG | AGAGCAGATGATAGTAGAG | NM_001031422 | This study |
| *PLRG1* | GCACAGTGAGATTAGATT | ATCATATTCAGGAGACATC | ENSGALT00000015067 | This study |
| *SYK* | GAACTGCTTGTGTAGATG | CTGTCACTCACTAGATGTA | NM_001031430 | This study |
| *LAPTM4B* | TGTTGTTGATGAAGGTTCTACT | GTGGAGTGGTGAGAGGTA | ENSGALT00000043979 | This study |
| *CASR* | ATGAGATGTATTCGTGATTGA | ACTGACTATGGTGTGGAA | ENSGALT00000023275 | This study |
| *FAM219A* | GTCAGTTCATAGTTCATTC | AATTGTGTTGTGTTAGTC | ENSGALT00000009350 | This study |
| *TDRD7* | ATACGAATGTCAGAGTAAC | TCCTGGTGAAGTAGATAG | NM_001305104 | This study |
| *ARID1A* | GTCTCAGCAGCCAATCAA | TTCTTCATCAGGAACTTCAACT | ENSGALT00000000486 | This study |
| *CSMD1* | TCCAGAGGTTATGAGGTT | TCCAAGTTAAGTTAGCAAGA | ENSGALT00000026359 | This study |
| *RNLS* | AGTGTCAGAAGTGGAGAT | GAGAGGTTGAGTATGAAGAAT | NM_001199599 | This study |
| *CACNA1B* | GCAGACAAGTAGACCATCA | ACCACTCGTTGACCAATC | NM_204293 | This study |
| *HAPLN1* | CTGTATGGTGTCTACTGTT | AGTAAGTAAGAAAGGTTTATCAC | NM_205482 | This study |
| *RUNX1* | GCTTTCAATTTACCGATG | TCTGGAAGTGATGTATGA | NM_205227 | This study |
| *TRIB1* | ATATTCAGACCAGGAGAC | TTAGCAGAAGAAGGAACT | ENSGALT00000026313 | This study |
| *PREPL* | GCTAATAGACTTGGAGATGT | GCTGTATTGATTGAACTGAAT | ENSGALT00000044298 | This study |
| *OTOG* | TGACTGTTAGGATGACTA | GTATGTGTTGATGTTGTAG | ENSGALT00000010023 | This study |
| *FEM1C* | TAACTTCAGTCCACTTCAC | AGATAGCAGTAACTTGTAGAG | ENSGALT00000013361 | This study |
| *RALGPS1* | CTTCAGGCAGCATTATGG | GTAGCGTTGTAAGATGTTCA | ENSGALT00000001330 | This study |
| ENSGALT00000045092 | GTGGCTTCTTGGATTCTT | CATTGGAGGTAACATTGC | ENSGALT00000045092 | This study |
| *PARPBP* | AGGCTCTGTCCAATCTTA | TTGTTGCTGTTGTTGAATC | NM_001030763 | This study |
| *NSD1* | AACATTGAGATGCCAGAA | CAGACGACTTCCTTGTAG | ENSGALT00000004699 | This study |
| *ACTB* (β-actin) | CTCCCTGATGGTCAGGTCAT | AGACAGCACTGTGTTGGCATA | NM_205518.1 | [5] |
| CR1-B | AAGGCGCAGGTAGAACTGAA | AGTGAAGACCGAGGCAAAGA | CR1-B | This study |
| CR1-C | GGAGATGCTCCTCAAGCAGT | GCTCCAGAGCCTCATACCTG | CR1-C | This study |
| CR1-D | CAGGGAGGTGATATGGGAGA | GTGGGGTACCATGTCAAAGG | CR1-D | This study |
| CR1-E | TAACCAACATGGCTTCACCA | GTGGTCCATCCATCAAATCC | CR1-E | This study |
| CR1-F | CAGTGGCAGGGAAAGACAAT | TACAAGTCCTGCCAGCTCCT | CR1-F | This study |
| CR1-G | CTTTGATCATGGGGCAGTTT | TCTCTGGTGAGCCCTGTTTT | CR1-G | This study |
| CR1-H | GCCAGTGCTTGTAGGAGAGG | CCTTTTGAGTGGCAGTAGCC | CR1-H | This study |
| *PRKD1* | GCCTGTCAAACGTTTCCTTAAC | GTCGCCCAATGTAGGATTGT | NM_001031201 | This study |
| *LRP8* | GCGATAAGGACCAGTTCCAG | CCACCTATCTGGGATGCAGT | NM_205186 | This study |
| *SMARCD3* | TCCCGTGCTTGTTTTTAACC | TGCAGTGCGTTTTCTTGTTG | ENSGALT00000021298.4 | This study |
| *SEMA5A* | ATTTGCTGGAGGTGGGAAG | CACGACTGCACTGAGACCAT | ENSGALT00000021302.4 | This study |
| *NTRK2* | ATTGCCTGGTTGGTGAGAAC | CCAGGCTCCAGACATCACTT | NM_205231 | This study |
| *GDPD5* | TACTACGAGCCGCAGCTATG | TCATTGTGAACTTCCCACCA | NM_001305150 | This study |
| *KATNB1* | CACACGACACCCATTGAGAG | TGGAAATCAAGGCTGCAGAT | NM_001030559 | This study |
| *SEMA4D* | CAGCACAGATGGAGAAGACG | GCATATCAATCTGGCCTTGA | NM_001293102 | This study |
| *PAX6* | CGGCAGAAGATCGTGGAACTCG | GCACTCTCGTTTATACTGCGCTAT | NM_205066 | [6] |

# References

1. Murtagh F, Legendre P: **Ward’s Hierarchical Agglomerative Clustering Method: Which Algorithms Implement Ward’s Criterion?** *Journal of Classification* 2014, **31**(3):274-295.

2. Kuleshov MV, Jones MR, Rouillard AD, Fernandez NF, Duan Q, Wang Z, Koplev S, Jenkins SL, Jagodnik KM, Lachmann A *et al*: **Enrichr: a comprehensive gene set enrichment analysis web server 2016 update**. *Nucleic Acids Res* 2016, **44**(W1):W90-97.

3. Lee SI, Lee BR, Hwang YS, Lee HC, Rengaraj D, Song G, Park TS, Han JY: **MicroRNA-mediated posttranscriptional regulation is required for maintaining undifferentiated properties of blastoderm and primordial germ cells in chickens**. *Proc Natl Acad Sci U S A* 2011, **108**(26):10426-10431.

4. Kim TH, Yun TW, Rengaraj D, Lee SI, Lim SM, Seo HW, Park TS, Han JY: **Conserved functional characteristics of the PIWI family members in chicken germ cell lineage**. *Theriogenology* 2012, **78**(9):1948-1959.

5. Chang KW, Huang NA, Liu IH, Wang YH, Wu P, Tseng YT, Hughes MW, Jiang TX, Tsai MH, Chen CY *et al*: **Emergence of differentially regulated pathways associated with the development of regional specificity in chicken skin**. *BMC Genomics* 2015, **16**:22.

6. Azuma N, Tadokoro K, Asaka A, Yamada M, Yamaguchi Y, Handa H, Matsushima S, Watanabe T, Kohsaka S, Kida Y *et al*: **The Pax6 isoform bearing an alternative spliced exon promotes the development of the neural retinal structure**. *Hum Mol Genet* 2005, **14**(6):735-745.
